# AutoMH: Automatically Create Evolutionary Metaheuristic Algorithms Using Reinforcement Learning

**DOI:** 10.3390/e24070957

**Published:** 2022-07-10

**Authors:** Boris Almonacid

**Affiliations:** Global Change Science, Puerto Varas 5550000, Chile; boris.almonacid@globalchange.science

**Keywords:** machine learning, reinforcement learning, optimisation, metaheuristic, evolutionary metaheuristic, high-level data driven metaheuristics, metaheuristic generation, online learning, search trajectory networks

## Abstract

Machine learning research has been able to solve problems in multiple domains. Machine learning represents an open area of research for solving optimisation problems. The optimisation problems can be solved using a metaheuristic algorithm, which can find a solution in a reasonable amount of time. However, the time required to find an appropriate metaheuristic algorithm, that would have the convenient configurations to solve a set of optimisation problems properly presents a problem. The proposal described in this article contemplates an approach that automatically creates metaheuristic algorithms given a set of optimisation problems. These metaheuristic algorithms are created by modifying their logical structure via the execution of an evolutionary process. This process employs an extension of the reinforcement learning approach that considers multi-agents in their environment, and a learning agent composed of an analysis process and a process of modification of the algorithms. The approach succeeded in creating a metaheuristic algorithm that managed to solve different continuous domain optimisation problems from the experiments performed. The implications of this work are immediate because they describe a basis for the generation of metaheuristic algorithms in an online-evolution.

## 1. Introduction

The use of metaheuristic algorithms has become an approach widely used to solve a variety of optimisation problems, such as optimisation problems in the fields of health, logistics, agriculture, mining, space, robotics, etc. In the last decade, the diversity of metaheuristic algorithms has grown widely [1], with a great diversity of components, routines, selectors, internals, and especially a great variety of parameters. This diversity leads to different difficulties, such as, for example, being able to find a specific configuration of parameters for a specific type of optimisation problem. This describes a situation that induces and generates challenges in choosing a metaheuristic algorithm correctly. Various strategies have been adopted to minimise the effort of manual configurations. One area is machine learning, specifically in reinforcement learning [2], where various advances have been made. For example, the implentation of a general method to reformulate reinforcement learning problems as optimisation tasks and then application of the particle swarm metaheuristic algorithm to find optimal solutions [3]. Solutions to solve the vehicle routing problem [4] include, feature selection [5], the design of a plane frame [6], or resource allocation problems [7]. Other approaches include Learn-heuristics [8], Q-Learning [9], Meta-learning [10], and Hyper-heuristic [11,12], which provide diverse perspectives on optimisation problems. In [13], multi-agent reinforcement learning is proposed, which allows for an upgrade in the reinforcement learning area, which generally uses a single agent.

In algorithm generation, there is an approach that uses the construction of a centralised hybrid metaheuristic cooperative strategy to solve optimisation problems [14]. Another approach employs a set of instructions to create a set of machine learning algorithms in real-time [15]. A basis for understanding the scope of these approaches can be found in [16], which provides the taxonomy of combinations with metaheuristics, mathematical programming, constraint programming, and machine learning. Open problems and the area’s current status can be found in [17,18].

This research focuses on contributing within the area of High-Level Data-Driven Metaheuristics on the topic of Metaheuristic Generation by Reinforcement Learning described in [17]. Specifically this research is under the following the flow of taxonomy High-Level Data Driven Metaheuristics → Metaheuristic Generation → Online Learning → Reinforcement Learning → *AutoMH framework*. This research aims to find, through an evolutionary generation process based on reinforcement learning, the best metaheuristic algorithm(s) that solve the set of optimisation problems given by the user. The main benefits expected from this work are as follows:

- Design a framework based on reinforcement learning that allows, through an online evolution process, to automatically generate evolutionary metaheuristic algorithms capable of solving a portfolio of optimisation problems in a viable manner.
- Incorporate flexibity into the framework design to add diverse components such as operators, intensification functions, and exploration functions.
- Contribute to the area of machine learning for optimisation, specifically in the integration of reinforcement learning to solve optimisation problems.

The rest of this paper is structured as follows: In Section 2, the proposed design and the formalisation of its components are detailed. In Section 3, the tests performed and their results are detailed. Finally, Section 4 concludes and provides guidelines for future work.

## 2. AutoMH Framework

This section presents the design of the *AutoMH framework* and the main components that make up the extended model of reinforcement learning (RL). Moreover, how the AutoMH components interact with the template of the evolutionary metaheuristic algorithm in the internal modification of the template structure with new instructions or modifications is explored.

### 2.1. General Reinforcement Learning Model

**Figure 1:**
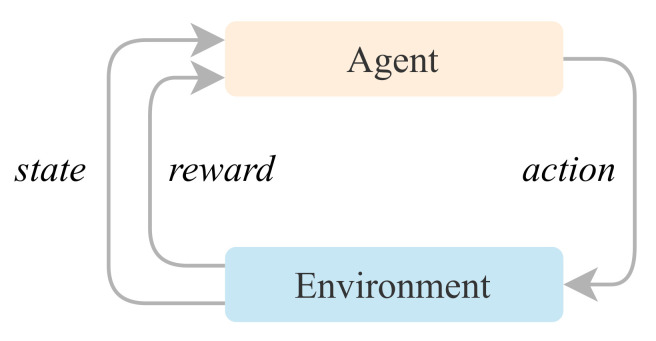
General reinforcement learning model.

A general reinforcement learning model determines what actions an agent should choose to maximise the objective in a given environment. An overview of RL can be seen in Figure 1. A general RL model consists of two components:

- A *Learning Agent* as the component that we want to train and learn to make decisions.
- An *Environment* that consists of the environment in which the *Learning Agent* interacts. The environment contains the possible constraints and rules.

Between the agent and environment components, there is a relationship that feeds back and has the following connections:

- An *Action* is chosen from a set of possible actions that the learning *Agent* can take at a given time.
- A *State* that corresponds to a set of indicators updated from the environment of how the various elements composing it is functioning.
- A *Reward* arises for each action performed by the *Learning Agent*. This reward can be a prize or a penalty. This information guides the *Learning Agent* toward identification of correct or incorrect behaviour.

### 2.2. Proposed AutoMH Framework

**Figure 2:**
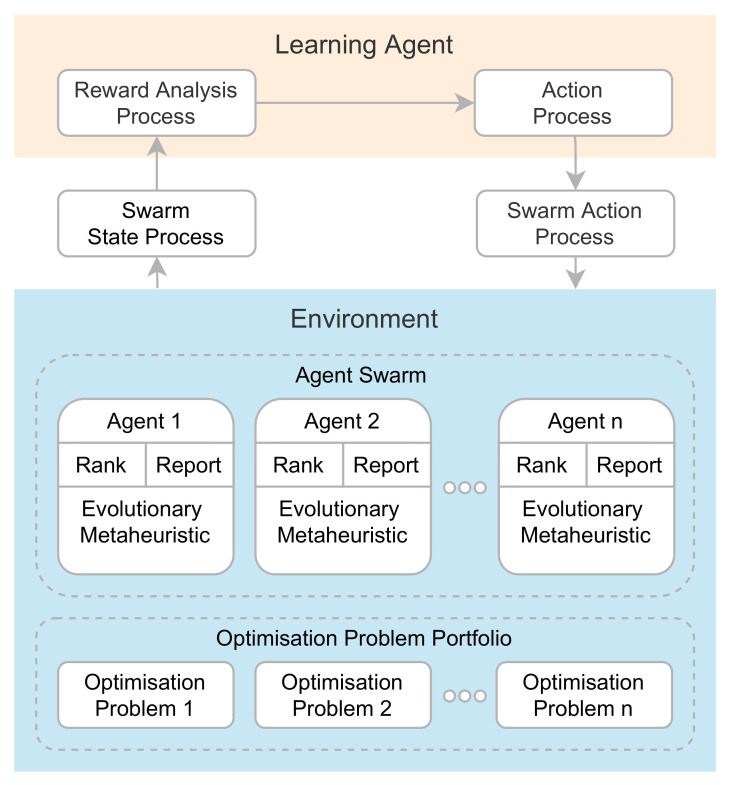
Proposed *AutoMH framework* for the automatic creation of metaheuristics.

The proposed *AutoMH framework* automatically aims to create metaheuristic evolutionary algorithms using a reinforcement learning modification. Metaheuristic evolutionary algorithms are contained within non-intelligent agents. The agents are immersed in the environment and are in charge of carrying out the benchmark that consists of solving a portfolio of optimisation problems by executing the metaheuristic evolutionary algorithm that the agent contains. During its execution, the *AutoMH framework* is constantly searching for new metaheuristic algorithms, finding suitable and unsuitable algorithms in each episode to solve the portfolio. The suitable algorithms can be kept in the following episode while the unsuitable algorithms are modified. At the end of the execution, the *AutoMH framework* has as its output the best agent in the environment with an evolutionary metaheuristic algorithm capable of finding the best solutions for a portfolio of optimisation problems. Figure 2 details the main parts of the framework architecture. It consists of two essential components of a reinforcement learning system: the *Learning Agent* and the *Environment*.

The *Learning Agent* is in charge of analysing the information from the environment and determining and performing actions on non-intelligent agents. The *Learning Agent* is formally specified in Definition 1.

**Definition** **1.** *Learning Agent: The learning agent bears the function of analysing the data generated by the environment through the Reward Analysis Process and taking actions that will affect through a set of actions the internal behaviour of each agent in the swarm of agents through the Action Process.*

The *Environment* is composed of a set of non-intelligent agents. Each agent has a base template of a metaheuristic algorithm which evolves in each episode by modifying its structure. This template is initially empty and is later transformed by adding instructions, removing instructions or maintaining instructions from its structure through the modifications made by the *Learning Agent*. The general components of the *Environment* are defined in Definitions 2–4.

**Definition** **2.** *Environment: The environment is composed of two elements:*

- *A set of non-intelligent agents $A=\{A_{1},A_{2},\ldots ,A_{n}\}$.*
- *An optimisation problem portfolio $P=\{p_{1},p_{2},\ldots ,p_{m}\}$ that must be executed by the non-intelligent agents.*

**Definition** **3.** *An Agent $A_{i}$ is defined by the three-tuple $A_{i}=\langle M,Q,S\rangle$, where:*

- *An Evolutionary Metaheuristic Algorithm M, which is an empty structure named template τ. This structure is modified at run-time by the swarm action process by adding, modifying, or removing instructions.*
- *A Qualification Q corresponds to a variable that indicates the value of the rank assigned to the agent.*
- *A State S that corresponds to a report with a set of data structures in which the optimisation tests results are stored. The stored data correspond to a set of summaries with fitness, and solution for each optimisation problem. Moreover, the fitness value information for each iteration.*

**Definition** **4.** *A continuous optimisation problem p is defined by minimise the objective function f(x) subjecttol≤x≤u, where $x=[x_{1},x_{2},\ldots ,x_{d}]$, d is a positive value ≥ 2 that represents the dimension of the optimisation problem, $l=[l_{1},l_{2},\ldots ,l_{d}]$, and $u=[u_{1},u_{2},\ldots ,u_{d}]$ are the lower bounds and the upper bounds of the corresponding variables in x, which define the feasible domain of the problem p.*

A thorough explanation of the components and their interactions are detailed in Section 2.3, Section 2.4, Section 2.5, Section 2.6, Section 2.7 and Section 2.8.

### 2.3. Instruction

An instruction *I* is an ordered grouping of elements with the objective of producing a change in the value of a variable. An instruction is made up of four elements: a variable, an assignment operator, an operator, and a function. The composition of an instruction is detailed in the Equation (1). Where, from right to left: $q(x_{t})$ is the function that is applied using the current value of the variable $x_{t}$ in order to generate a new value, Δ is the operator that will be applied with the value of the variable $x_{t}$ and with the value obtained by applying the function $q(x_{t})$. Additionally, the symbol ← is the assignment operator for a new value that will be assigned in $x_{t+1}$, and the symbol *t* indicates the iteration number. Formally, an instruction is determined by Definition 5.

(1) $$\begin{array}{ccccc} x_{t+1} & \leftarrow & x_{t} & \Delta & q(x_{t}) \end{array}$$

**Definition** **5.** *An instruction I is composed by a variable x, one generic instruction operator $O=\{\Delta_{k}\left(x\right)\;|\;k\in K\}$, and one intensification function $H=\{h_{i}\left(x\right)\;|\;i\in L\}$, or one exploration function $G=\{g_{j}\left(x\right)\;|\;j\in J\}$, where K={1…m}, J={1…n}, and L={1…l}. The values of m, n, and l are determined by the initial information of the system.*

Additionally, instructions can derive into instruction types such as an *exploration instruction* $I_{\varepsilon }$, which is defined by function (2), or an *intensification instruction*$I_{\gamma }$ that is defined by function (3).

(2) $$I_{\varepsilon }\left(x,\Delta_{k},g_{j}\right)=x\;\Delta_{k}\;g_{j}\left(x\right)$$

(3) $$I_{\gamma }\left(x,\Delta_{k},h_{i}\right)=x\;\Delta_{k}\;h_{i}\left(x\right)$$

**Definition** **6.** *An operator* Δ *is a mathematical symbol that indicates that a specific operation must be performed on a variable and an exploration function g(x) or an intensification function h(x).*

#### Instruction Component Feature Considerations

Instruction must be executed atomically; this means that the calculation of the variable’s new value should not integrate new components such as operations, procedures, or additional functions of those already defined in Equation (1). The complex procedures that modify the variable are built through instructions using the *AutoMH framework*. The format of consecutive instructions to generate complex processes is described in Equation (4).

(4) $$\begin{array}{c} x_{t+1}\;\;=\;\;x_{t}\;\;\Delta \;\;q\left(x_{t}\right)\\ x_{t+2}\;\;=\;\;x_{t+1}\;\;\Delta \;\;q\left(x_{t+1}\right)\\ x_{t+3}\;\;=\;\;x_{t+2}\;\;\Delta \;\;q\left(x_{t+2}\right)\\ \;\;\;\;\;\;\;\;\;\;\;\;\;\;\;\;\ldots \\ x_{t+n}\;\;=\;\;x_{t+n-1}\;\;\Delta \;\;q\left(x_{t+n-1}\right) \end{array}$$

The operators must allow for the performing of an operation between the value of the variable *x* and the function q(x) output value. Through this operation, a new value of the variable *x* is obtained. The variable’s new value can be decreased, increased, or unchanged.

A function represents a simple and defined behaviour. Additionally, in the functions g(x) or h(x), the input parameter of the value of the variable *x* is optional.

An intensification function h(x) must always exhibit the same behaviour each time it is used; that is, it must always return the same result when given the same parameters. In addition, it must not contain random components. An example of a function h(x) is a function that returns the value of the trigonometric function sine; if we also consider a delta addition operator, then the instruction is composed of the following structure $x_{t+1}\leftarrow x_{t}\;+\;sin\left(x_{t}\right)$. If we instantiate the variable *x* with the value 1.3, the result of the instruction described in Equation (5) has an increase in the value of the variable *x* given the value provided by function $sin(x_{t})$.

(5) $$\begin{array}{ccc} x_{t+1} & = & x_{t}\;+\;sin\left(x_{t}\right)\\ x_{t+1} & = & 1.3\;+\;sin(1.3)\\ 2.26355818542 & = & 1.3\;+\;0.96355818541 \end{array}$$

An exploration function g(x) must exhibit stochastic behaviour each time it is used; it must always return a random value. An example of a function g(x) is a function that returns a random value obtained over a continuous interval [0,1]; if we also consider a subtraction delta operator, then the instruction is composed with the following structure $x_{t+1}\leftarrow x_{t}-U∼\left(0,1\right)$. If we instantiate the variable *x* with the value 1.3, the result of the instruction described in Equation (6) will decrease the value of the variable *x*.

(6) $$\begin{array}{ccc} x_{t+1} & = & x_{t}-U\left(0,1\right)\\ x_{t+1} & = & 1.3\;-\;U(0,1)\\ 0.9679 & = & 1.3\;-\;0.3321 \end{array}$$

### 2.4. Evolutionary Metaheuristic Algorithm

An evolutionary metaheuristic algorithm *M* is a template that changes in each episode depending on the decisions made by the *Learning Agent* through the *Action Process*. Modifications to its structure are performed at run-time through *Swarm Action Process*. Formally, *M* is determined by Definition 7.

**Definition** **7.** *An evolutionary metaheuristic algorithm is defined by the 4-tuple M=〈τ,E,Γ,δ〉, where: τ is a metaheuristic template that is composed by*Initial*,*step*,*end*, and*run*functions, E is an sequence of exploration functions $E=[g_{1},g_{2},\ldots ,g_{n}]$,* Γ *is an sequence of intensification functions $\Gamma =[h_{1},h_{2},\ldots ,h_{n}]$, and δ is an set of operators $\delta =\{\Delta_{1},\Delta_{2},\ldots ,\Delta_{n}\}$.*

- The Initial function is in charge of initialising the variables of the optimisation problem. Initialisation is carried out using one or more exploration instructions. Subsequently, the current fitness is calculated and the solution is stored.
- The Step function is the main core of the template. In this function the main modifications are made in the evolutionary metaheuristic algorithm. Actions are carried out such as adding, modifying or deleting instructions both of the type of exploration instructions, as well as intensification instructions. Subsequently, the new fitness of the solution is calculated, and the new fitness and solution is stored in the event that it is better than the previous one.
- The End function is executed when the end condition of the metaheuristic algorithm ends. Its function is to extract the solution found and its associated fitness.
- The Run function has the purpose of executing the Initial, Step and End functions.

**Figure 3:**
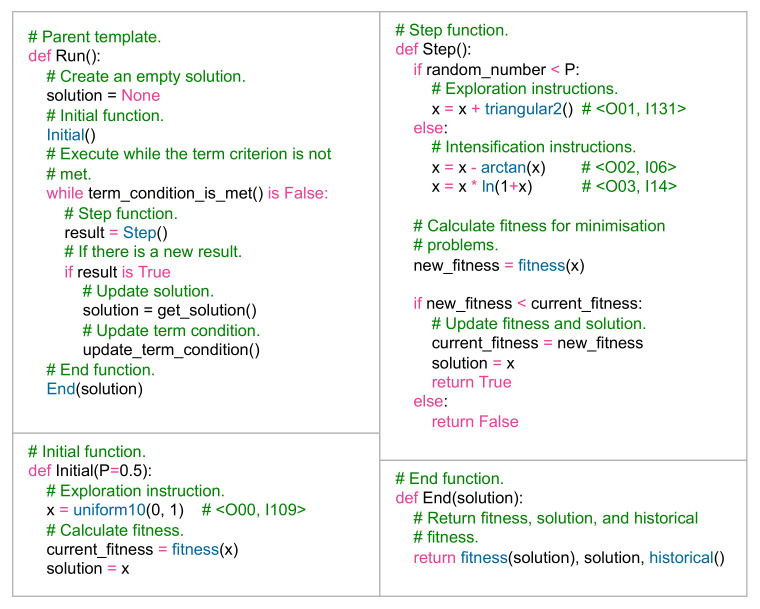
Metaheuristic template τ.

Figure 3 describes an example of a template τ that has already been modified by the *Learning Agent*. The Run function is the main template. The Initial function has a single instruction that is composed of the operator None with the code O00, and by the uniform10(0, 1) function with the code I109. The Step function is composed of an exploration instruction 〈O01,I131〉, and two intensification instructions 〈O02,I06〉 and 〈O03,I14〉. The End function returns the fitness, solution, and the historical fitness (it is the fitness saved in each iteration).

At the end of the execution of the metaheuristic algorithm *M*, it outputs the fitness, the solution and the historical fitness of a problem *p*. The definition of the output is described in Equation (7). In which, from left to right, f(x) is the value of the objective function of a problem *p*, the solution is the array of variables, and the historical fitness is an array where the fitness values of each iteration are stored, the array values must satisfy $f{\left(x\right)}_{i}\geq f{\left(x\right)}_{i+1}$.

(7) $$Output=\{f\left(x\right),\left[x_{1},x_{2},\ldots ,x_{dimension}\right],\left[f{\left(x\right)}_{1},f{\left(x\right)}_{2},\ldots ,f{\left(x\right)}_{max\_iteration}\right]\}$$

The output corresponds to an observation $O_{state}$ of the behaviour of metaheuristic algorithm *M* when solving a problem *p*. The definition is described in Equation (8) and corresponds to the order of the components in Equation (7), where $R^{+}=\left\{x\in R|x\geq 0\right\}$, *l* and *u* are the lower and upper bounds on R of variable *x*, and l<u. Within the definition, the *space-size* can be observed, which is directly related to the domain that the variables have in a problem *p*. Observation $O_{state}$ has a number of 1+dimension+max_iteration elements.

(8) $$\begin{array}{cccc} \; & fitness & \; & historical\;\;fitness\\ O_{state}= & \{\overset{︷}{R^{+}},\; & \underset{︸}{\left[{\left[l,u\right]}_{1},{\left[l,u\right]}_{2},\ldots ,{\left[l,u\right]}_{dimension}\right]},\; & \overset{︷}{\left[R_{1}^{+},R_{2}^{+},\ldots ,R_{max\_iteration}^{+}\right]}\}\\ \; & \; & space-size \end{array}$$

A numerical example of an observation *O* is described in Equation (9), where the array of variables *x* has a dimension value of 2, *l* is −10.0, *u* is 10.0, and the value of max_iteration is 10.

(9) O={0.0001,[0.0002,0.0003],[8,7.3,4.4,3.2,1.003,0.734,0.11,0.021,0.003,0.0001]}

### 2.5. Swarm State Process

The *Swarm State Process* consists of a process to collect the observation $O_{state}$ generated by the swarm of non-intelligent agents when solving a problem *p* using an algorithm *M*. The purpose of this process is to be able to have all the *states* of the non-intelligent agents of the environment in a single structure.

The first step is to build a matrix that contains the *partial-state* of the swarm, that is, that incorporates the *state* information of a single non-intelligent agent. Each cell of this matrix must contain a single observation of an execution of an algorithm *M* in solving a problem *p*. This matrix is defined as $A=(O_{ij})$ (see Equation (10)), where i∈{1,…,m}; j∈{1,…,k}, *m* is the total number of optimisation problems, *k* is the number of executions that an algorithm *M* solves a problem *p*.

(10) $$Agent_{partial-state}=A_{ps_{i}}=\begin{array}{cc} & \begin{array}{cccc} E_{1} & E_{2} & \cdots & E_{k} \end{array}\\ \begin{array}{c} P_{1}\\ P_{2}\\ \vdots \\ P_{m} \end{array} & \left[\begin{array}{cccc} O_{1,1} & O_{1,2} & \cdots & O_{1,k}\\ O_{2,1} & O_{2,2} & \cdots & O_{2,k}\\ \vdots & \vdots & \ddots & \vdots \\ O_{m,1} & O_{m,2} & \cdots & O_{mk} \end{array}\right] \end{array}$$

Finally, the second step is to group all the *partial-states* of all the non-intelligent agents in the swarm to obtain the total *state*. The *state* is defined by Equation (11).

(11) $$State=\{A_{ps_{1}},A_{ps_{2}},\ldots ,A_{ps_{n}}\}$$

### 2.6. Reward Analysis Process

The objective of the *Reward Analysis Process* is to rank each agent in the swarm. Whether a non-intelligent agent has obtained a good or bad ranking is related to whether it has obtained a good or bad reward according to its results in solving the portfolio of problems when using its algorithm *M*.

In order to obtain the ranking of the non-intelligent agents, a procedure must be performed to transform the information from continuous values in R that the state has, to discrete values in N. This initial approximation procedure consists of extracting the fitness of each observation, and each execution of a problem *p* when using an algorithm *M* (see Equation (12)). Subsequently, to approximate this observation, a calculation is made using the mean according to Equation (13). Equations (12) and (13) describe the process only for problem $P_{1}$; however, this process must be carried out with each problem that the agent has.

(12) $$\begin{array}{ccccc} \; & E_{1} & E_{2} & \; & E_{k}\\ P_{1} & O_{1,1} & O_{1,2} & \cdots & O_{1,k}\\ \; & ↓ & ↓ & \; & ↓\\ P_{1} & f{\left(x\right)}_{1,1} & f{\left(x\right)}_{1,2} & \cdots & f{\left(x\right)}_{1,k} \end{array}$$

(13) $$mean_{i,j}=\frac{f{\left(x\right)}_{1,1},f{\left(x\right)}_{1,2},\ldots ,f{\left(x\right)}_{1,k}}{k}$$

The set of approximations using the mean fitness is represented by the matrix $Q=(q_{i,j})$ (See Equation (14)), where i∈{1,…,m},j∈{1,…,n}, *m* is the number of optimisation problems, and *n* is the number of agents in the swarm. Each cell $q_{i,j}$ has been calculated using the procedure of Equations (12) and (13).

(14) $$Q=\begin{array}{cc} & \begin{array}{cccc} A_{1} & A_{2} & \cdots & A_{n} \end{array}\\ \begin{array}{c} P_{1}\\ P_{2}\\ \vdots \\ P_{m} \end{array} & \left[\begin{array}{cccc} q_{1,1} & q_{1,2} & \cdots & q_{1,n}\\ q_{2,1} & q_{2,2} & \cdots & q_{2,n}\\ \vdots & \vdots & \ddots & \vdots \\ q_{m,1} & q_{m,2} & \cdots & q_{mn} \end{array}\right] \end{array}$$

The second part of the approximation process consists of performing a series of operations that comprises:

1. The assignment of ranges is conducted using the data provided by the matrix *Q*. The method used is the minimum method (competition method), which in order to perform ranking to each value, the minimum of the ranges that would have been assigned is assigned to all tied values. The ranking result is stored in matrix $R=(r_{i,j})$ (See Equation (15)), where: i∈{1,…,m},j∈{1,…,n}, *m* is the number of optimisation problems, and *n* is the number of agents in the swarm.
  (15) $$R=\begin{array}{cc} & \begin{array}{cccc} A_{1} & A_{2} & \cdots & A_{n} \end{array}\\ \begin{array}{c} P_{1}\\ P_{2}\\ \vdots \\ P_{m} \end{array} & \left[\begin{array}{cccc} r_{1,1} & r_{1,2} & \cdots & r_{1,n}\\ r_{2,1} & r_{2,2} & \cdots & r_{2,n}\\ \vdots & \vdots & \ddots & \vdots \\ r_{m,1} & r_{m,2} & \cdots & r_{mn} \end{array}\right] \end{array}$$
2. The minimum method is performed for each row of the matrix *Q*, which considers that each problem bears its own ranking among all the agents. The ranking result for each row will be stored in matrix *R*.
3. A sum of each column in the matrix *R* is performed. Each sum will correspond to the final ranking value for each agent in the swarm. The values of each sum are stored in a vector *S* where each value of cell $S\in N^{n}$.

(16) $$S=\begin{array}{c} \begin{array}{cccc} A_{1}\; & A_{2} & & \;A_{n} \end{array}\\ \left[\begin{array}{cccc} \sum_{i=1}^{m}r_{i,1} & \sum_{i=1}^{m}r_{i,2} & \cdots & \sum_{i=1}^{m}r_{i,n} \end{array}\right] \end{array}$$

### 2.7. Action Process

The *Action Process* takes the information generated by the *Reward Analysis Process* and performs a swarm modification procedure. To do this, we define a matrix $A_{m,n}$ (See Equation (17)), where the first row corresponds to the values calculated from Equation (16), and the second row $A_{2,i}$ represents the actions to be assigned to each value $s_{i}↔A_{1,i}$.

(17) $$A_{m,n}=[\begin{array}{cccc} s_{1,1} & s_{1,2} & \cdots & s_{1,n}\\ a_{2,1} & a_{2,2} & \cdots & a_{2,n} \end{array}]$$

An action can have only one of the following cases: N↔ None, M↔ Modify, or R↔ Restart. The case None action means that the agent will not have any modifications made to its metaheuristic algorithm *M*. The case Modify action means that the agent can carry out modifications in the structure of its metaheuristic algorithm *M*. In the case of Restart action, the template τ of the non-intelligent agent will be initialised with random instructions.

The steps to calculate the actions are as follows:

1. Sort the swarm agents from the best ranking to the worst ranking (See Equation (18)).

(18) $$\begin{array}{cc} S_{i}= & \left[\begin{array}{cccccccccc} 8 & 6 & 13 & 9 & 7 & 7 & 4 & 7 & 10 & 6 \end{array}\right]\;\mathrm{unsorted}\\ S_{i}= & \left[\begin{array}{cccccccccc} 4 & 6 & 6 & 7 & 7 & 7 & 8 & 9 & 10 & 13 \end{array}\right]\;\mathrm{sorted}\mathrm{for}\mathrm{minimisation}\mathrm{problems}\\ S_{i}= & \left[\begin{array}{cccccccccc} 13 & 10 & 9 & 8 & 7 & 7 & 7 & 6 & 6 & 4 \end{array}\right]\;\mathrm{sorted}\mathrm{for}\mathrm{maximisation}\mathrm{problems} \end{array}$$

2. The partitions for the array $S_{i}$ are calculated and included in the matrix *A*. In this step there can be two cases:
  - The *standard case* is when there is a single best ranking (See Equation (19)). The best ranking is marked with the None action, and the remaining number of rankings are divided in two, marking one part with the Modify action, and the other part with the Restart action. This case also applies when all agents have the same ranking value.
    (19) $$\begin{array}{c} A_{m,n}=[\begin{array}{ccc} \begin{array}{c} 4\\ a_{2,1} \end{array} & |\begin{array}{ccccc} 6 & 6 & 7 & 7 & 7\\ a_{2,2} & a_{2,3} & a_{2,4} & a_{2,5} & a_{2,6} \end{array} & |\begin{array}{cccc} 8 & 9 & 10 & 13\\ a_{2,7} & a_{2,8} & a_{2,9} & a_{2,10} \end{array} \end{array}]\\ A_{m,n}=[\begin{array}{ccc} \begin{array}{c} 4\\ N \end{array} & |\begin{array}{ccccc} 6 & 6 & 7 & 7 & 7\\ M & M & M & M & M \end{array} & |\begin{array}{cccc} 8 & 9 & 10 & 13\\ R & R & R & R \end{array} \end{array}] \end{array}$$
  - An *alternative case* is when there are multiple best rankings (See Equation (20)). That is, the same ranking value exists in other agents. The first best ranking must be chosen in the group of the best rankings and marked with the None action, and the remaining amount of the group are to be divided in two, marking one part with the Modify action, and the other part with the Restart action. Divide the remaining amount of rankings in two, marking one part with the Modify action, and the other part with the Restart action.
    (20) $$\begin{array}{c} \;S_{i}=\left[\begin{array}{cccccccccc} 4 & 4 & 4 & 4 & 4 & 4 & 8 & 9 & 10 & 13 \end{array}\right]\\ A_{m,n}=[\begin{array}{ccccc} \begin{array}{c} 4\\ a_{2,1} \end{array} & |\begin{array}{ccc} 4 & 4 & 4\\ a_{2,2} & a_{2,3} & a_{2,4} \end{array} & |\begin{array}{cc} 4 & 4\\ a_{2,5} & a_{2,6} \end{array} & |\begin{array}{cc} 8 & 9\\ a_{2,7} & a_{2,8} \end{array} & |\begin{array}{cc} 10 & 13\\ a_{2,9} & a_{2,10} \end{array} \end{array}]\\ A_{m,n}=[\begin{array}{ccccc} \begin{array}{c} 4\\ N \end{array} & |\begin{array}{ccc} 4 & 4 & 4\\ M & M & M \end{array} & |\begin{array}{cc} 4 & 4\\ R & R \end{array} & |\begin{array}{cc} 8 & 9\\ R & R \end{array} & |\begin{array}{cc} 10 & 13\\ R & R \end{array} \end{array}] \end{array}$$

### 2.8. Swarm Action Process

**Figure 4:**
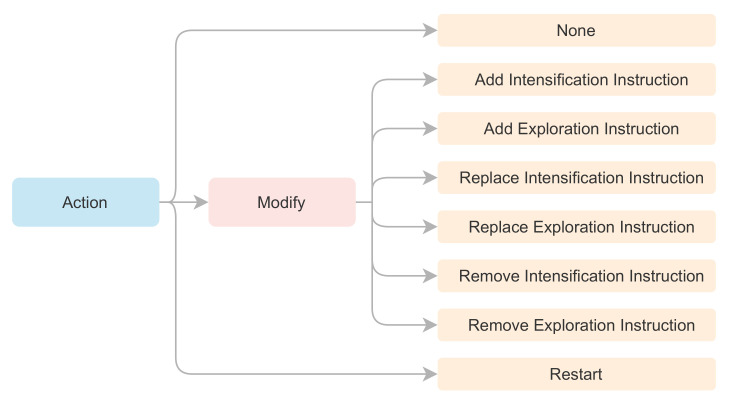
Summary of the allowed action-space for the *Learning Agent*.

The *Swarm Action Process* has the function of modifying the agents of the swarm that bear the Modify case. To carry out the modifications, each agent obtains a random integer employing a discrete uniform distribution U{1,6}. The value obtained will correspond to a type of action that will modify the metaheuristic algorithm’s instruction structure. The allowed modifications are Add, Replace, and Remove for instructions Intensification, and Exploration. Figure 4 shows the allowed set of actions for the *Learning Agent*, giving an action-space of eight movements.

**Figure 5:**
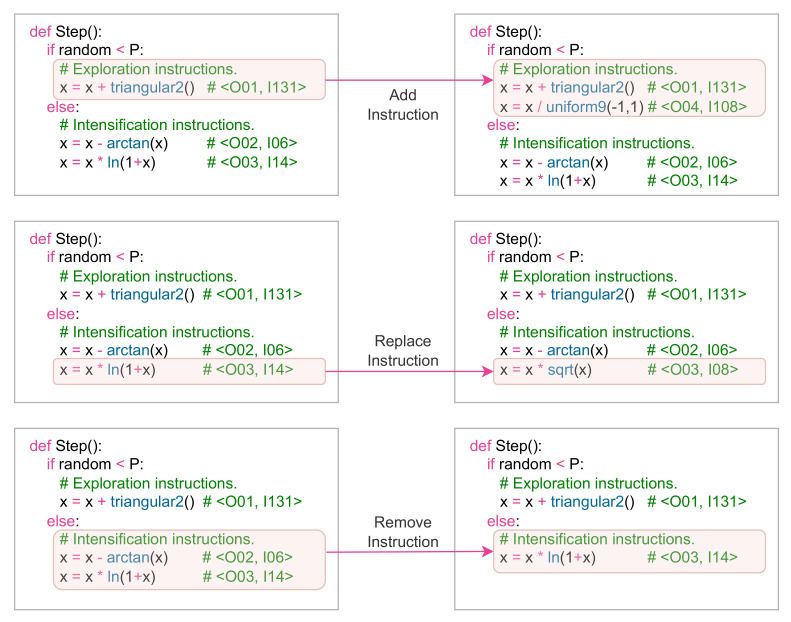
Example of modifications in the structure of the metaheuristic algorithm.

Figure 5 shows the three types of modifications that are made in the metaheuristic algorithm *M*. From these modifications, the agent can repeat the optimisation tests to observe whether the structure changes generate better or poorer results.

**Figure 6:**
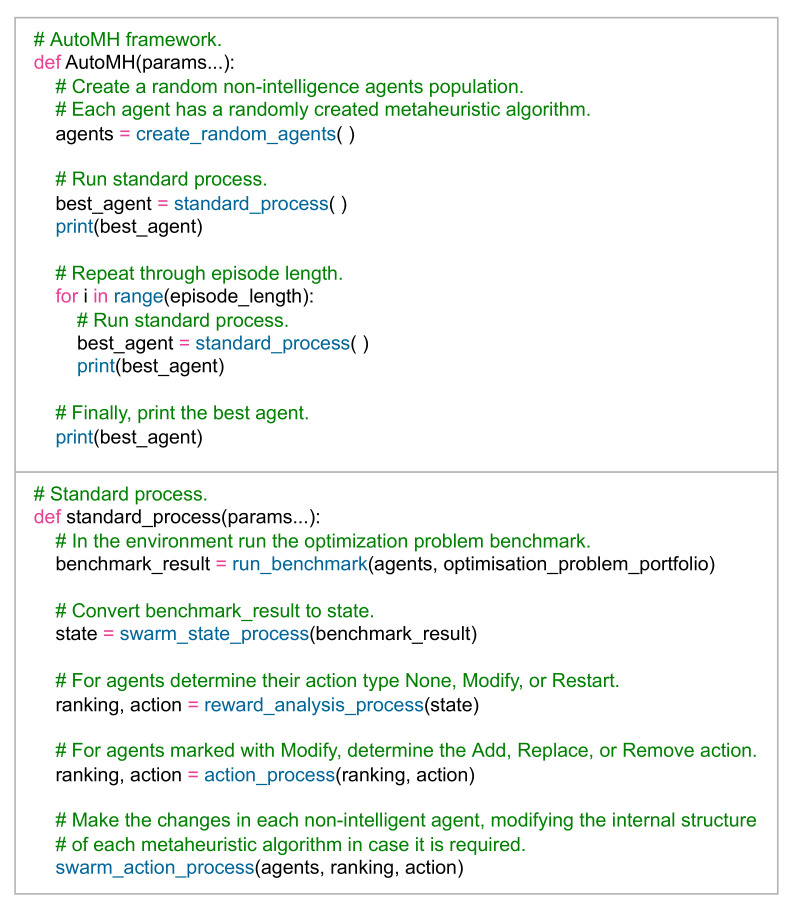
*AutoMH framework* pseudocode.

In summary, Figure 6 describes the pseudocode of the *AutoMH framework*.

## 3. Experiments

**Figure 7:**
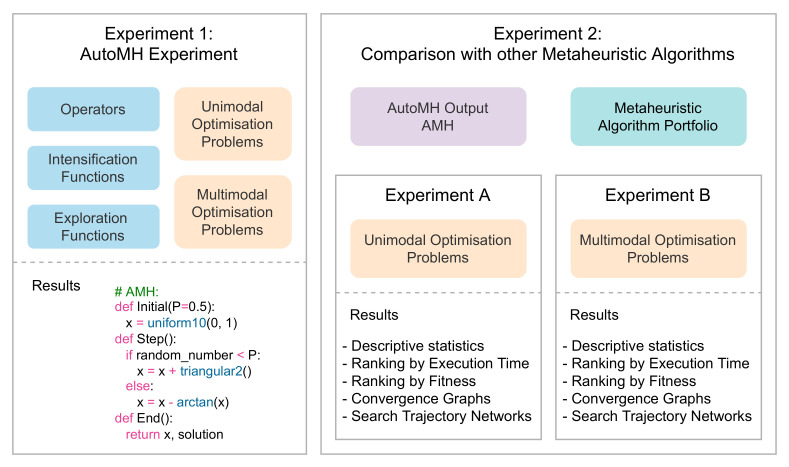
Overview of experiments.

This section focuses on presenting the test design of the *AutoMH framework* and the results obtained in various tests. Figure 7 describes a global view of the two experiments carried out. Section 3.4 describes the details and results of executing Experiment 1: AutoMH Experiment. Section 3.6 and Section 3.7 describe the details and results of executing Experiment A and Experiment B that make up Experiment 2: Comparison with other Metaheuristic Algorithms.

The environment used in the experiments is described in Section 3.1. The optimisation problems used in the experiments are described in Section 3.2. The operators, the intensification functions and the exploration functions used in experiment Section 3.4 are described in Section 3.3. The metaheuristic algorithms used to perform comparative tests of *Experiment A* and *Experiment B* are described in Section 3.5, Section 3.6 and Section 3.7.

### 3.1. General Environment

The experiment was developed in Python language version 3.9.6, running on an MSI P65 Creator 9SE laptop with Intel Core I7 9750H CPUs @ 2.60 Ghz, 16 GB RAM, and Windows 10 Pro OS build 19041.685.

### 3.2. Optimisation Problem Dataset

The experiments will focus on solving a portfolio composed of 13 continuous optimisation problems. These optimisation problems are divided into two groups of problems. The first group of problems is composed of seven unimodal optimisation problems. These problems are described in Table 1 and are numbered as P1, P2, P3, P4, P5, P6, and P7. The second group of problems is composed of six multimodal optimisation problems. These problems are described in Table 2 and are numbered as P8, P9, P10, P11, P12, and P13.

### 3.3. Portfolio of Operators, Intensification Functions, and Exploration Functions

The experiment uses a set of portfolios composed of operators, intensification functions, and exploration functions. These operators and functions will be used to build by executing the *AutoMH framework* intensification instructions (see Equation (3)) and exploration instructions (see Equation (2)). The constructed list of instructions is used to complete the metaheuristic template τ and obtain a new metaheuristic algorithm. A description of the portfolios are provided below.

- A portfolio comprising a list of operators identified as O00, O01, O02, O03, and O04. These operators are described in Table 3.
- A portfolio containing a list of intensification functions is described in Table 4. These functions are a set of essential mathematical functions. These enhancement functions are individualised from identifier I01 to identifier I14.
- Two portfolios are composed of a list of exploration functions.
  - The first portfolio contains a list of random number generating functions, such as uniform, beta, and triangular functions. These functions are described in Table 5 and individualised from identifier I100 to identifier I121.
  - The second portfolio contains a list of functions that return a constant. These constants come from The On-Line Encyclopedia of Integer Sequences (OEIS) [25] and have been chosen considering constants known in the literature. These functions are individualised in Table 6 from identifier I200 to identifier I212.

### 3.4. Experiment 1: AutoMH Experiment

In this experiment, the *AutoMH framework* aims to find an algorithm capable of obtaining good results solving a set of two groups of continuous optimisation problems. Table 7 describes the parameters used in the *AutoMH* experiment, such as *AutoMH* setting (T01 to T03), the metaheuristic template τ configuration (T04 to T05), the dimension of the optimisation problem (T06), the list of initial, exploration, intensification and operator instructions (T07 to T12), and the restriction of the minimum and the maximum number of instructions that the generated metaheuristic could have.

#### Experiment Results

During the execution of the experiment using the *AutoMH framework*, 403,000 executions were performed (10 agents × 100 episodes × 31 executions × 13 problems), and 40,300,000 evaluations of objective functions were performed (10 agents × 100 episodes × 31 executions × 13 problems × 100 iterations). The *Action Process* during the 100 episodes has executed the *standard case* a total of 94 times, consequently the *alternative case* has been executed a total of 6 times.

**Figure 8:**
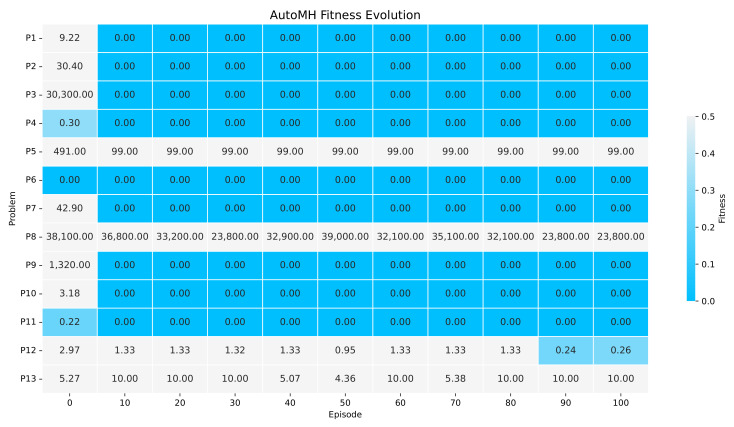
The Figure showing the evolution of fitness during the 100 episodes of execution of *AutoMH framework*. The figure summarises the episodes 10 by 10.

Figure 8 shows the evolution of fitness during the execution of *AutoMH framework*. The y-axis indicates the continuous optimisation problem, and the x-axis indicates the AutoMH episode. Each column indicates the results of the best agent in that episode, in which each cell indicates the mean value of the 31 executions carried out by that agent for a continuous optimisation problem. According to Figure 8, a set of relevant information can be extracted. During the initial episode (episode 0), *AutoMH framework* has initialised with one problem with a global fitness value and two problems with a fitness less than 0.4. By episode 10, AutoMH has already obtained a global fitness value for problems F1, F2, F3, F4, F6, F7, F9, F10, and F11, giving a total of eight, and problem F10 with a lower fitness value to 0.0001. From episode 20 to episode 80, there are no improvements for new problems, maintaining the eight problems with global fitness, however, attempts to improve problems F8, F12, and F13 can be observed. Finally, for the episodes from 90 to 100, an improvement has been achieved in the F12 problem with a fitness lower than 0.3.

**Figure 9:**
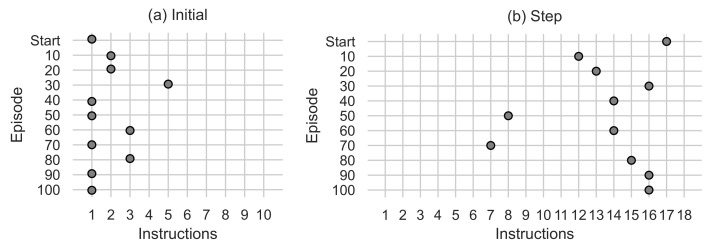
(**a**) Shows the number of instructions used in the Initial function. (**b**) Shows the number of instructions used in the Step function.

**Figure 10:**
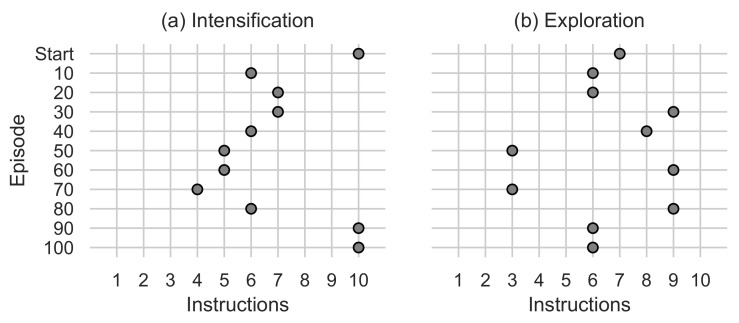
(**a**) Shows the number of intensification instructions used by the Step function. (**b**) Shows the number of exploration instructions used by the Step function.

Figure 9 and Figure 10 show variations in the number of instructions used in the algorithm generated by the best agent in the episode. The y-axis indicates the episode, and the x-axis indicates the instructions used. Figure 9a indicates the number of initial instructions used in the Initial function. The number of instructions used in the generated algorithms ranges from 1 to 5, using the maximum number of instructions at episode 30, and ending at episode 100 with a single initial instruction. Figure 9b indicates the number of steps and scan instructions used in the Step function. The number of instructions used in the generated algorithms is 7 to 16, using 16 instructions in episode 100. The 16 instructions are divided into 10 intensification instructions and 6 exploration instructions visualised in Figure 10.

The metaheuristic algorithm generated by the *AutoMH framework* can be extracted as a result at the end of episode 100 in the best ranked non-intelligent agent of the swarm. As a note, this algorithm is generated in episode 95 and has remained in the best ranking without another algorithm generated exceeding it. From the output of this agent, the following instruction tuple sequences can be extracted:

**Initial**       (〈O00,I110〉).
**Intensification**  (〈O01,I07〉,〈O01,I06〉,〈O04,I01〉,〈O03,I06〉,〈O01,I14〉,〈O01,I03〉, 〈O02,I09〉,〈O04,I03〉〈O02,I07〉,〈O02,I04〉).
**Exploration**   (〈O00,I100〉,〈O04,I201〉,〈O03,I212〉,〈O04,I202〉,〈O01,I110〉, 〈O02,I112〉).

**Figure 11:**
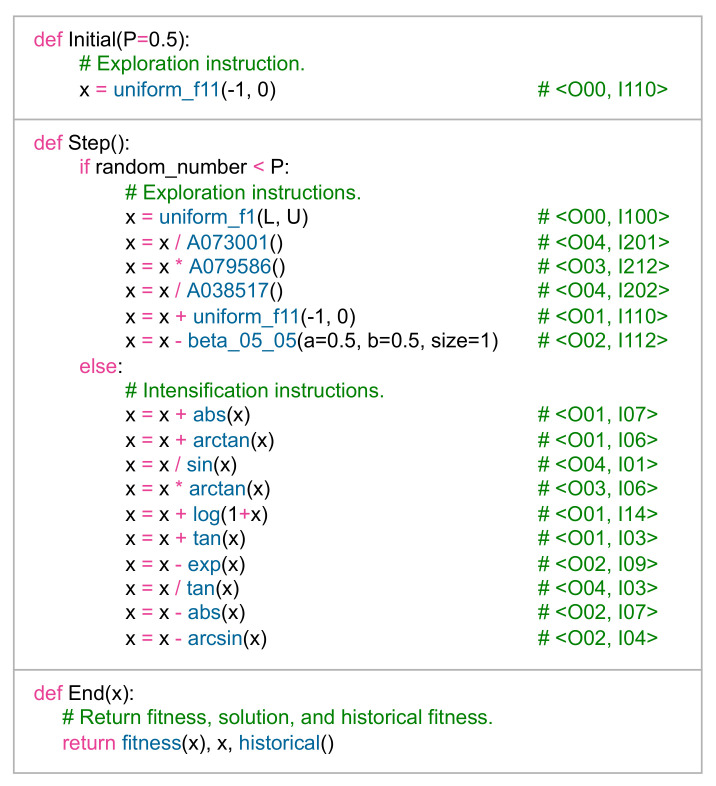
Best algorithm found when running the *AutoMH framework*.

These instructions are the code fragments with which the metaheuristic algorithm is generated as output from the *AutoMH framework*. The equivalent pseudocode of the three sequences can be seen in Figure 11. During the rest of the manuscript, the generated metaheuristic algorithm of Figure 11 will be referred to as AMH.

### 3.5. Experiment 2: Comparison with Other Metaheuristic Algorithms

The objective of *Experiment 2* is to carry out tests comparing the performance of the AMH algorithm obtained when executing *Experiment 1*. Two experiments will be conducted:

- *Experiment A*: The first group of tests comprises a set of unimodal optimisation problems. These problems are described in Table 1 and numbered as P1, P2, P3, P4, P5, P6, and P7. The results of this experiment are developed in Section 3.6.
- *Experiment B*: The second group of tests comprises a set of multimodal optimisation problems. These problems are described in Table 2 and numbered as P8, P9, F10, P11, P12, and P13. The results of this experiment are developed in Section 3.7.

The conditions of the experiment are indicated below:

- Experiments *A* and *B* will carry out the execution of 15 metaheuristic algorithms. These algorithms are listed below:
  - AMH, which is the algorithm that automatically generates the *AutoMH framework* through *Experiment 1* in Section 3.4.
  - Bat Algorithm (BAT) [26,27].
  - Cuckoo Search (CS) [28,29].
  - Differential Evolution (DE) [30].
  - FireFly Algorithm (FFA) [31].
  - Genetic Algorithm (GA) [32].
  - Grey Wolf Optimiser (GWO) [33].
  - Harris Hawks Optimization (HHO) [34].
  - Jaya algorithm (JAYA) [35].
  - Moth-Flame Optimization (MFO) [36].
  - Multi-Verse Optimiser (MVO) [37].
  - Particle Swarm Optimisation (PSO) [38].
  - Sine Cosine Optimization Algorithm (SCA) [39].
  - Salp Swarm Algorithm (SSA) [40].
  - Whale Optimization Algorithm (WOA) [41].
- Each algorithm is performed 31 times for each optimisation problem in *Experiment A* and *Experiment B*.
- As a termination condition, each algorithm is stopped after completing 100 iterations.
- The parameters of each algorithm are the default values from the Evolopy framework [42]. A population value of 6 has been used for the swarm intelligence algorithms, except for the AMH algorithm, which is a single population.
- It is considered that an algorithm has managed to reach the optimal global value when during the iterations or at the end of them, the fitness values are less than the tolerance value $1.00\times 10^{-8}$.

Each experiment will arrange the results through various perspectives.

*A summary of descriptive statistics results*: The results are described by means of a table that shows the quantitative performance indicators of mean fitness and standard deviation. This summary describes the results obtained by each metaheuristic algorithm for each optimisation problem. Complementary results that include a nonparametric multiple comparisons test are described in the Appendix B.

*A set of box plots*: The purpose is to visualise the fitness results of each algorithm in each optimisation problem. The visualisation of the results of each metaheuristic algorithm will be conditioned to appear in the visualisation if the total sum of its fitness is less than the value of 1.00 × 10^10^ otherwise, they will not be considered in the box plot.

*A run-time summary*: A summary of the execution times that the algorithms have taken to solve each optimisation problem’s executions.

*A ranking summary*: A visual summary shows a ranking of the algorithms when solving the portfolio of problems. This ranking considers the indicators of mean and execution time. The ranking summary is represented by the matrix $R=(r_{ij})$ (see Equation (21)), where i∈{1,…,m};j∈{1,…,n}, *m* is the total number of optimisation problems, and *n* is the total number of the metaheuristic algorithms. Each row indicates the ranking for a single optimisation problem.

(21) $$R=\begin{array}{cc} & \begin{array}{cccc} A_{1} & A_{2} & \cdots & A_{n} \end{array}\\ \begin{array}{c} P_{1}\\ P_{2}\\ \vdots \\ P_{m} \end{array} & \left[\begin{array}{cccc} r_{1,1} & r_{1,2} & \cdots & r_{1,n}\\ r_{2,1} & r_{2,2} & \cdots & r_{2,n}\\ \vdots & \vdots & \ddots & \vdots \\ r_{m,1} & r_{m,2} & \cdots & r_{mn} \end{array}\right] \end{array}$$

In order to obtain the results of the matrix *R*, the data must be obtained and grouped into a data matrix *D*. Formally, the data matrix can be defined as $D=({\langle F;T\rangle }_{i,j})$ (see Equation (22)), where *F* is the mean value of the fitness value of the 31 runs of an optimisation problem using a metaheuristic algorithm, *T* is the sum of the times of the 31 executions of an optimisation problem using a metaheuristic algorithm, i∈{1,…,m};j∈{1,…,n}, *m* is the total number of optimisation problems, and *n* is the total number of the metaheuristic algorithms.

(22) $$D=\begin{array}{cc} & \begin{array}{cccc} A_{1}\;\; & \;A_{2} & \;\cdots & \;\;A_{n} \end{array}\\ \begin{array}{c} P_{1}\\ P_{2}\\ \vdots \\ P_{m} \end{array} & \left[\begin{array}{cccc} {\langle F;T\rangle }_{1,1} & {\langle F;T\rangle }_{1,2} & \cdots & {\langle F;T\rangle }_{1,n}\\ {\langle F;T\rangle }_{2,1} & {\langle F;T\rangle }_{2,2} & \cdots & {\langle F;T\rangle }_{2,n}\\ \vdots & \vdots & \ddots & \vdots \\ {\langle F;T\rangle }_{m,1} & {\langle F;T\rangle }_{m,2} & \cdots & {\langle F;T\rangle }_{m,n} \end{array}\right] \end{array}$$

An example of the ranking calculation for a problem can be seen in the Equation (23), where:

- Step 0: The data are available in the matrix *D*.
- Step 1: A first ranking is performed by ordering the algorithms considering the best fitness mean among the results of all the algorithms.
- Step 2: In the case of a tie in the value of the mean fitness between two or more algorithms, it should be considered that the algorithms are ordered according to the time in which the algorithms managed to execute the 31 executions of an optimisation problem. The algorithm with the shorter time will receive the best ranking, and the algorithm with the longer time will receive the worse ranking.
- Step 3: Finally, the ranking by mean and time is obtained.

(23) $$\begin{array}{ll} \mathrm{Step}\;0 & \;\begin{array}{ccccc} & A_{1} & A_{2} & A_{3} & A_{4}\\ D=P_{1} & [\langle 0.5;8.34\rangle & \langle 0.3;7.64\rangle & \langle 0.6;9.77\rangle & \langle 0.3;7.22\rangle ] \end{array}\\ \mathrm{Step}\;1 & \;\begin{array}{ccccc} & A_{1} & A_{2} & A_{3} & A_{4}\\ D=P_{1} & [\langle 0.5;8.34\rangle & \langle 0.3;7.64\rangle & \langle 0.6;9.77\rangle & \langle 0.3;7.22\rangle ] \end{array}\\ & \begin{array}{ccccc} \mathrm{rank}\;\mathrm{by}\;\mathrm{mean} & \;A_{1}\; & A_{2}\; & A_{3}\; & A_{4}\\ \;R=P_{1} & [2\; & 1\; & 3 & 1] \end{array}\\ \mathrm{Step}\;2 & \;\begin{array}{ccccc} & A_{1} & A_{2} & A_{3} & A_{4}\\ D=P_{1} & [\langle 0.5;8.34\rangle & \langle 0.3;7.64\rangle & \langle 0.6;9.77\rangle & \langle 0.3;7.22\rangle ] \end{array}\\ & \begin{array}{ccccc} \mathrm{rank}\;\mathrm{by}\;\mathrm{time} & \;A_{1} & \;A_{2} & \;A_{3} & \;A_{4} \end{array}\\ & \begin{array}{cc} \;R=P_{1} & \left[\begin{array}{cccc} 2 & 1 & 3 & 1\\ \; & \;(\mathrm{longer}\;\mathrm{time})\; & & \left(\mathrm{shorter}\;\mathrm{time}\right) \end{array}\right] \end{array}\\ \mathrm{Step}\;3 & \;\mathrm{rank}\;\mathrm{by}\\ & \begin{array}{ccccc} \;\mathrm{mean}\&\mathrm{time} & \;A_{1}\; & A_{2}\; & A_{3}\; & A_{4}\\ \;R=P_{1} & \;[3\; & 2\; & 4\; & 1] \end{array} \end{array}$$

*A set of Convergence Graphs*: The idea is to visualise how the algorithms improve the fitness value during each iteration; in this way, it is possible to make a visual comparison of the convergence between several algorithms. Convergence graphs are built from the fitness results generated by each algorithm at each iteration. The construction of the convergence in this research is carried out through a numerical matrix of fitness. A numerical example of three runs is described in matrix (24). Each row represents a single run, and each column represents a single iteration. The executions are numbered as $\left\{E_{1},E_{2},E_{3}\right\}$ and the iterations are numbered as $\left\{I_{1},\ldots ,I_{8}\right\}$.

(24) $$\begin{array}{c} \begin{array}{cc} & \begin{array}{cccccccc} \;I_{1} & I_{2} & I_{3} & I_{4} & I_{5} & I_{6} & I_{7} & I_{8} \end{array}\\ \;\begin{array}{c} E_{1}\\ E_{2}\\ E_{3} \end{array} & \left[\begin{array}{cccccccc} 10 & \;4\; & \;4\; & \;3\; & \;2 & \;0 & \;0 & \;0\\ 8 & 3 & 3 & 3 & 2 & 1 & 0 & 0\\ 9 & 8 & 7 & 4 & 2 & 1 & 1 & 0 \end{array}\right] \end{array}\\ \begin{array}{ccccccccc} max & 9 & 8 & 7 & 4 & 2 & 1 & 1 & 0\\ average & 9 & 5 & \bar{4.7} & \bar{3.3} & 2 & \bar{0.7} & \bar{0.3} & 0\\ max & 8 & 3 & 3 & 3 & 2 & 0 & 0 & 0 \end{array} \end{array}$$

The three executions have eight iterations, starting at iteration $I_{1}$, which corresponds to the worst fitness until reaching iteration $I_{8}$, which corresponds to the best fitness. Using the matrix of fitness values, they are performed to determine the maximum fitness value, the mean fitness, and the minimum fitness for each column in the matrix. The maximum and minimum values plot the area of convergence, and the mean fitness indicates the mean convergence using a single array of values.

*A set of Search Trajectory Networks (STN)*: An STN is a directed graph defined as STN=G(N,E), where *N* is a node set and *E* is an edge set [43]. The purpose of the STN is to visualise the solutions generated by the optimisation algorithms in each iteration through a directed graph. Each node in the STN represents a location. A location represents a solution defined by a fitness value of the objective function. Each edge is directed to and connects two consecutive locations on the search path.

In this investigation, the STN is visualised through the deployment of a Fruchterman–Reingold design of force-directed graphs. The STN visualisation integrates the AMH algorithm by default, and two algorithms are chosen according to each experiment’s ranking. If the AMH algorithm is not displayed in a standard view, a subplot of the nodes with fitness values in the upper 25% percentile for the AMH algorithm is visible. For the visualisation of the STN, five executions have been taken as a sample for each metaheuristic algorithm.

### 3.6. Experiment 2 Results: Experiment A—Unimodal Optimisation Problems

This section describes and analyses the results of *Experiment A*. These results include statistical results and a view of these results from various perspectives such as box plots, runtime and fitness-based rankings, convergence plots, and Search Trajectory Networks plots.

#### 3.6.1. Statistical Results

Table 8 shows the results of *Experiment A* based on the quantitative indicators of mean and standard deviation.

**Figure 12:**
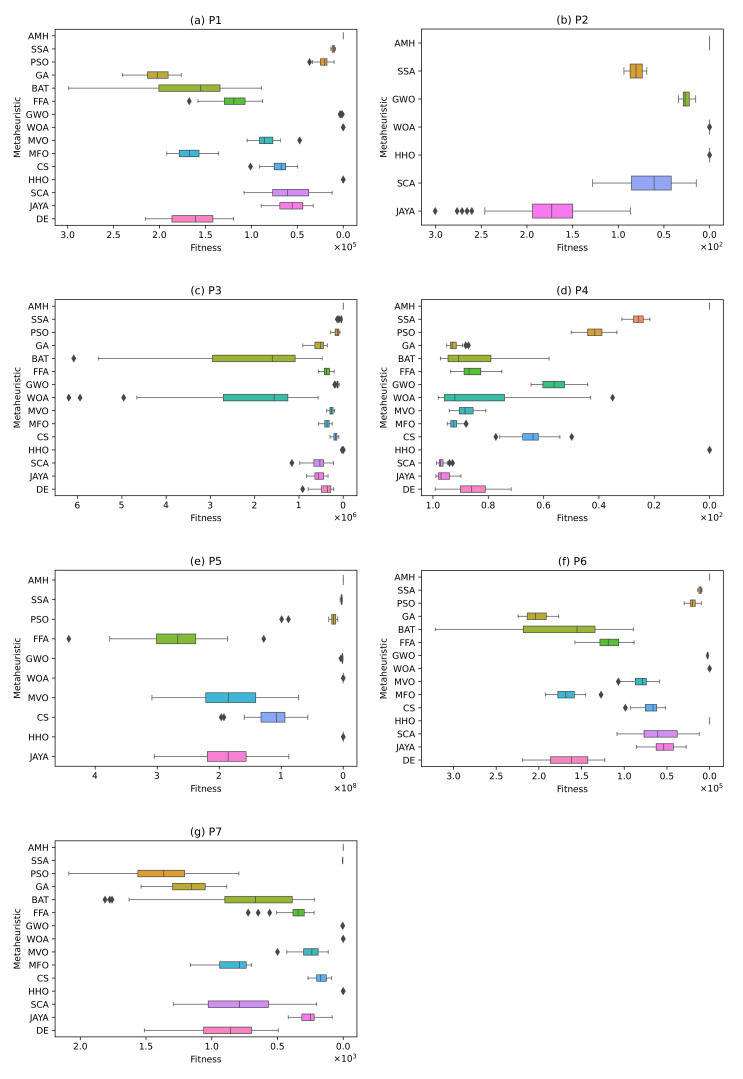
The box plots for problems P1, P2, P3, P4, P5, P6, and P7.

P1 Problem (Details in Definition A1): The optimal value was obtained by the AMH algorithm, with a mean value and standard deviation of 0.00 ± 0.00. The HHO algorithm displays a mean value and standard deviation of 1.96 × 10^11^ ± 1.03 × 10^10^. The BAT, CS, DE, FFA, GA, GWO, JAYA, MFO, MVO, PSO, SCA, SSA, and WOA algorithms have not performed well. These results can be visualised in Figure 12a, where it is observed that the algorithms closest to the optimal global value are the AMH and HHO algorithms. In addition, it is observed that these algorithms have a low dispersion in the data.

P2 Problem (Details in Definition A2): Visually, Figure 12b shows that the algorithms closest to the global optimum are AMH, WOA, and HHO. In addition, it is observed that these algorithms have a low dispersion in the data. If we consider the results of Table 8, the algorithm demonstrating better performance is the AMH algorithm, with a mean value of its fitness and a standard deviation of 0.00 ± 0.00. In a complementary way, the algorithms PSO, GA, BAT, FFA, MVO, MFO, CS, DE are not considered in Figure 12b, because the total sum of their fitness is greater than 1.00 × 10^10^.

P3 Problem (Details in Definition A3): Although Figure 12c visually shows that the algorithms closest to the global optimum are AMH, SSA, and HHO, the algorithm with better performance, however, is the AMH algorithm, with a mean fitness value of 0.00. Additionally, according to the data in Table 8, no other algorithm, except for AMH, achieved a fitness close to the global optimum or less than 1.00.

P4 Problem (Details in Definition A4): The algorithm with better performance is the AMH algorithm, with a mean fitness value of 0.00. The remaining algorithms did not reach good optimal values. These observations can be contrasted in Figure 12d.

P5 Problem (Details in Definition A5): In this problem, no algorithm demonstrated a mean fitness value less than the tolerance value 1.00 × 10^−8^. These observations can be contrasted in Figure 12e.

P6 Problem (Details in Definition A6): The algorithms that obtained an optimal value were the AMH and HHO algorithms, with a mean value and a standard deviation of 0.00. The BAT, CS, DE, FFA, GA, GWO, JAYA, MFO, MVO, PSO, SCA, SSA, and WOA algorithms did not perform well. These results can be contrasted in Figure 12f, where it can be seen that the algorithms closest to the optimal global value are the AMH and HHO algorithms.

P7 Problem (Details in Definition A7): The algorithms have managed to obtain an optimal value have been the AMH and HHO algorithms. The AMH algorithm has obtained an mean fitness value and a standard deviation of 0.00 ± 0.00, and the HHO algorithm has obtained an mean fitness value and a standard deviation of 1.65 × 10^−28^ ± 7.75 × 10^−28^. The BAT, CS, DE, FFA, GA, GWO, JAYA, MFO, MVO, PSO, SCA, SSA, and WOA algorithms have not performed well. These results can be seen in Figure 12g, where it can be seen that the algorithms closest to the optimal global value are the AMH and HHO algorithms.

#### 3.6.2. Execution Time

**Figure 13:**
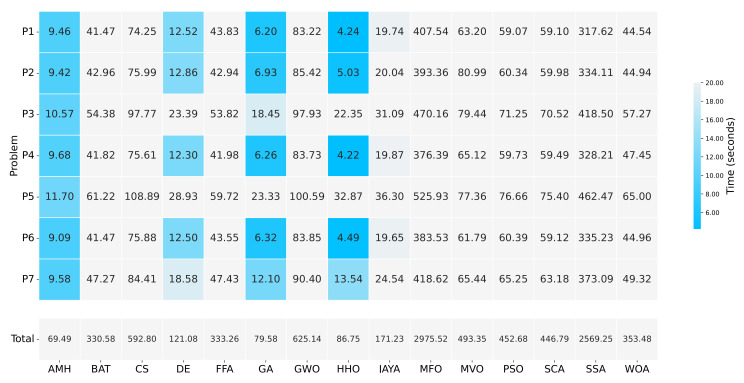
A summary of the execution times of Experiment A. The figure is composed of a matrix and a vector of values that represent a measurement in seconds. The matrix represents the results by a set of cells. The cells indicate the duration of the 31 executions in which each metaheuristic algorithm executed each optimisation problem. The vector represents the total sums for each column of values in the matrix. The calculation is performed by adding together the times of the problems P1, P2, P3, P4, P5, P6, and P7.

Figure 13 summarises the execution time in which each algorithm solved experiment A. It can be seen that the AMH algorithm obtained the best time with 69.49 s, the second-best time was obtained by the GA algorithm with 79.58 s, while the HHO algorithm obtained the third-best time with 86.75 s.

#### 3.6.3. Ranking

**Figure 14:**
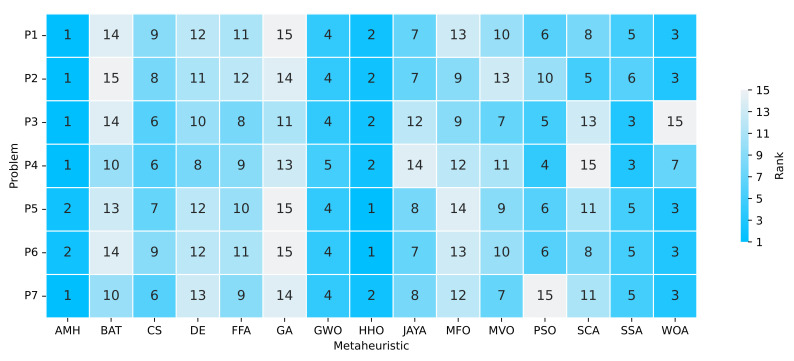
Summary of a ranking matrix between the algorithms in solving optimisation problems, considering mean fitness and execution time indicators. Each row represents the ranking among the 15 algorithms, ordered according to their performance at solving a problem P1, P2, P3, P4, P5, P6, or P7.

The ranking determines the position of the algorithm based on the indicators of the best mean fitness values and the shortest execution time. These results are displayed in Figure 14. If we only consider the algorithms that have obtained a ranking of 1, 2 or 3, we can extract the following observations:

- The AMH algorithm obtained five problems in rank 1 and two in rank 2.
- The HHO algorithm obtained two problems in rank 1 and five problems in rank 2.
- The WOA algorithm obtained five problems in rank 3.
- The SSA algorithm obtained two problems in rank 3.

From this information, we can deduce that the algorithm in first place with regard to the ranking is the AMH algorithm, the second algorithm in the ranking is the HHO, and the third algorithm in the ranking is the WOA.

#### 3.6.4. Search Trajectory Networks

The AMH, HHO, and WOA algorithms were chosen as they were the first three algorithms in the ranking described in Figure 14.

**Figure 15:**
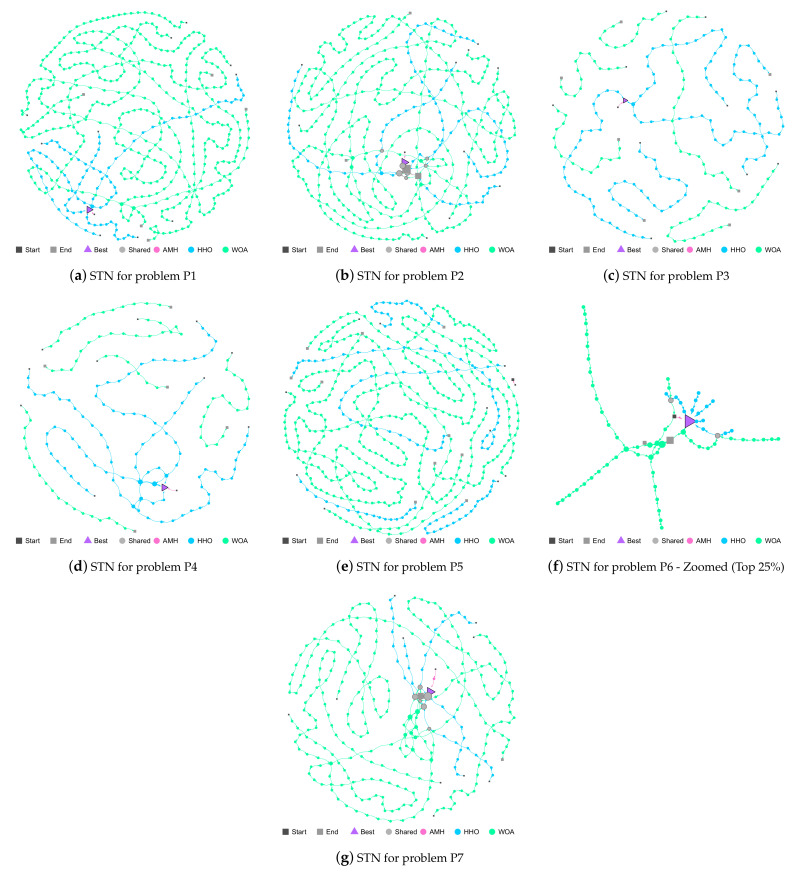
Figures (**a**–**g**) show the Search Trajectory Networks of the AMH, HHO, and WOA algorithms for problems P1, P2, P3, P4, P5, P6, and P7, respectively. The squares indicate the start and end locations of the algorithm executions. The triangle node is the best-found solution.The circles represent the nodes of algorithms AMH, HHO, and WOA. Each algorithm has a default colour for each circular node. If a circular node is shared by more than one algorithm, it is depicted in light grey.

P1 Problem: In Figure 15a, it can be seen that the trajectories of the AMH and HHO algorithms end in the best location (triangle node). In contrast, the WOA algorithm paths end at a different location.

P2 Problem: In Figure 15b, the five trajectories of the AMH algorithm have managed to reach the best location (triangle node), ending their location with a mean and standard deviation of 0.00 ± 0.00. The trajectory of the HHO and WOA algorithms have managed to approach a good location (square end node) with a mean and standard deviation of 2.16 × 10^−6^ ± 8.77 × 10^−6^ for the HHO algorithm and 7.91 × 10^−3^ ± 3.68 × 10^−2^ for the WOA algorithm; however, this location is insufficient with regard to acceptability as a good solution. It can also be seen that the HHO and WOA algorithms share several nodes in their trajectory.

P3 Problem: Figure 15c shows that the trajectories of the AMH algorithms end up in the best location (triangle node), with a mean and standard deviation of 0.00 ± 0.00. Regarding the HHO algorithm, two trajectories have failed to find a suitable solution, and three have managed to reach the best location (triangle node). For the WOA algorithm, it is observed that the five trajectories have performed an exploration in the search space but have not reached the best location.

P4 Problem: In Figure 15d, the five trajectories of the AMH algorithm have managed to reach the best location (triangle node), ending their location with a mean and standard deviation of 0.00 ± 0.00. The trajectory of the HHO algorithm visually also manages to reach the best location; however, when reviewing the values of the mean and standard deviation of 1.99 × 10^−6^ ± 7.53 × 10^−6^, it does not reach a better fitness at tolerance value 1.00 × 10^−8^. The five trajectories of the WOA algorithm have failed to come close to the best location.

P5 Problem: In this problem, no algorithm has managed to demonstrate a mean that represents values with the best fitness; therefore, the trajectories displayed in Figure 15e do not converge in the best location (triangle node).

P6 Problem: The trajectories of the AMH, HHO, and WOA algorithms are depicted in an enlarged display in Figure 15f. The trajectories of the AMH and HHO algorithms end in the best location (triangle node), with a mean and standard deviation of 0.00 ± 0.00. The HHO and WOA algorithms present two shared solutions (grey circle node). Two WOA trajectories reach the best location (triangle node); however, the other three do not reach a good position (large grey node). This observation can be contrasted with the mean and standard deviation with the obtained values of 1.58 ± 1.58.

P7 Problem: In Figure 15g, the trajectories of the AMH and HHO algorithms end in the best location (triangle node). This observation does not mean that the AMH and HHO algorithms have the same results, but rather that both are sufficient according to the tolerance value The trajectories of the AMH 1.00 × 10^−8^. The mean and standard deviation obtained for AMH was 0.00 ± 0.00, and 1.65 × 10^−28^ ±7.75 × 10^−28^ for HHO. The WOA algorithm was close to reaching the tolerance value 1.00 × 10^−8^, but still not enough, and achieved a close position (square node); this can be verified because its mean value and standard deviation are 1.49 × 10^−3^ ± 7.43 × 10^−3^. Finally, there are six solutions shared by the HHO and WOA algorithms.

#### 3.6.5. Convergence

**Figure 16:**
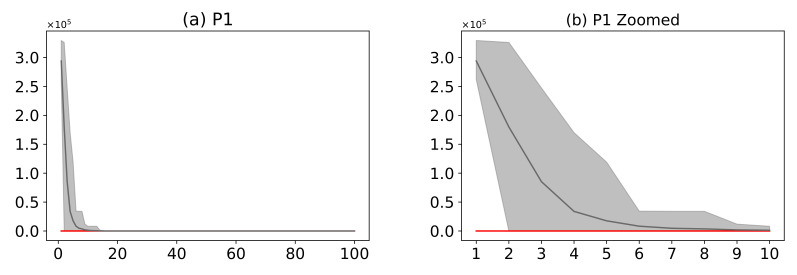

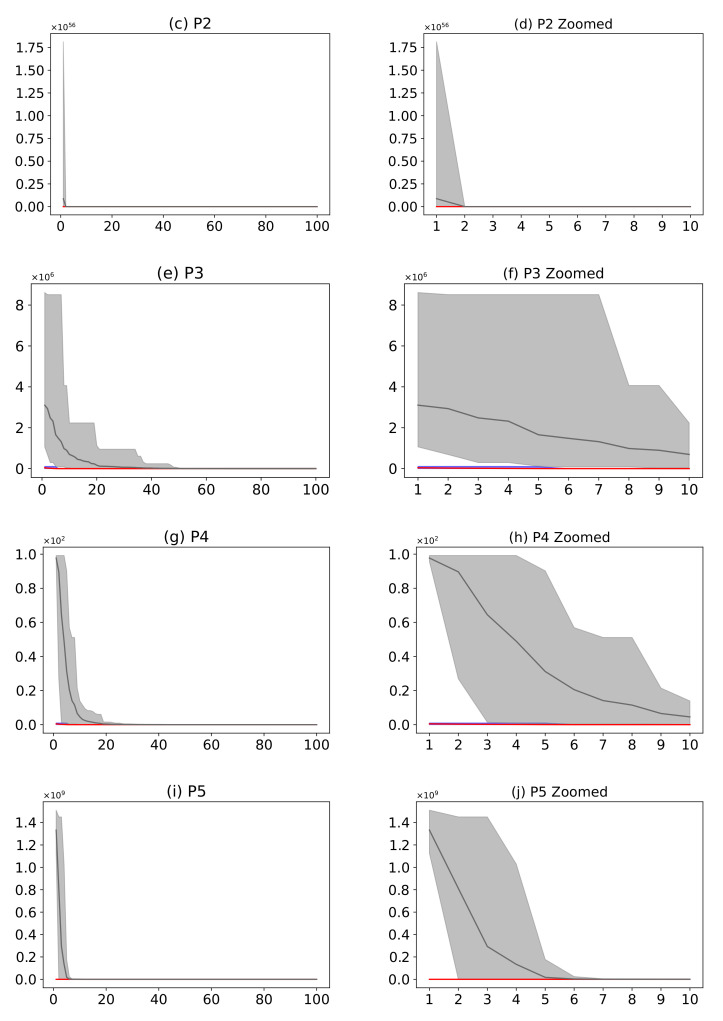

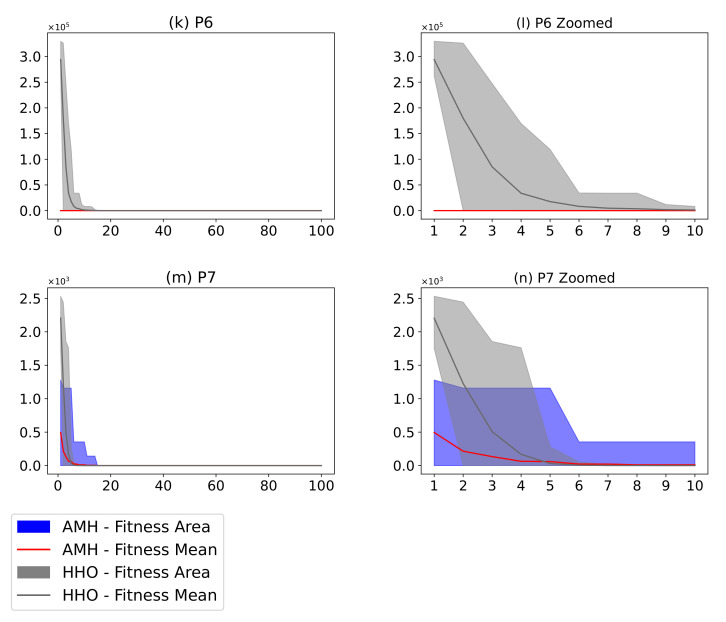
(**Plots a**,**c**,**e**,**g**,**i**,**k**,**m**) describe the convergence curves of the AMH and HHO algorithms for problems P1, P2, P3, P4, P5, P6, and P7; (**Plots b**,**d**,**f**,**h**,**j**,**l**,**n**) describe an enlarged view of the convergence curve from iteration 1 to 10. The x-axis indicates the number of iterations, and the y-axis indicates fitness. The areas represent the minimum and maximum fitness values obtained in each iteration for each algorithm. The lines represent the mean fitness value of each iteration. The information of the 31 executions is included.

Figure 16 shows a comparison of the AMH algorithm with the HHO algorithm. The AMH and HHO algorithms were chosen as they were the first and second algorithms in the ranking described in Figure 14. Based on the mean fitness value, it can be seen in Figure 16a,c,e,g,i,k,m that under 100 iterations, the AMH algorithm has a faster convergence compared to the HHO algorithm.

When considering the area of fitness for problems P1, P2, P3, P4, P5, and P6, the area of the AMH algorithm tends to be much smaller than the area of fitness of the HHO algorithm; providing partial evidence that the AMH algorithm tends to be much more robust than the HHO algorithm on this set of problems. The reason for this observation is because visually in each iteration, the minimum and maximum value of the fitness of the AMH algorithm tends to be lower in contrast to the minimum and maximum values of the fitness of the HHO algorithm. This robust observation is also observed between the first 10 iterations of Figure 16b,d,f,h,j,l. In the problem P7 of Figure 16m,n, the AMH algorithm tends to lose robustness but maintains a fast convergence according to the area.

### 3.7. Experiment 2 Results: Experiment B-Multimodal Optimisation Problems

This section describes and analyses the results of *Experiment B*. These results include statistical results and a view of these results from various perspectives such as box plots, runtime and fitness-based rankings, convergence plots, and Search Trajectory Networks plots.

#### 3.7.1. Statistical Results

Table 9 shows the results of *Experiment B* based on the quantitative indicators of mean and standard deviation.

**Figure 17:**
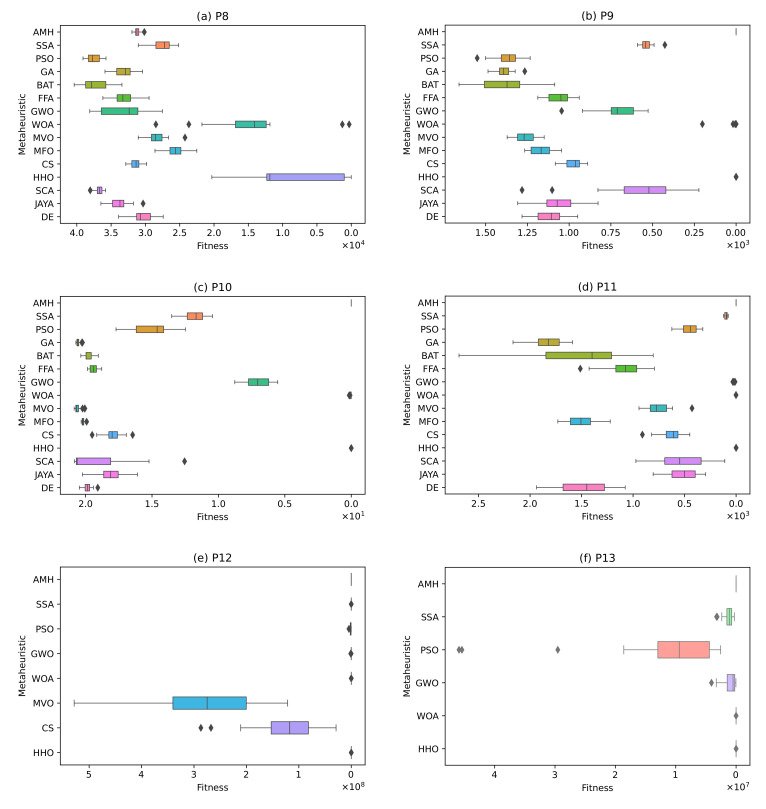
The box plots for problems P8, P9, P10, P11, P12, and P13.

P8 Problem (Details in Definition A8): In this problem, no algorithm achieved a value lower than the tolerance value 1.00 × 10^−8^. These data can be visually contrasted in Figure 17a.

P9 Problem (Details in Definition A9): The algorithms that have obtained an optimal value are the AMH algorithms with a mean fitness value and standard deviation of 0.00 ± 0.00, and the HHO algorithm with a mean and standard deviation of 1.60 × 10^−11^ ± 6.68. The BAT, CS, DE, FFA algorithms, GA, GWO, JAYA, MFO, MVO, PSO, SCA, SSA, and WOA did not perform well. These results can be visualised in Figure 17b, where it is observed that the algorithms closest to the global optimal value are the AMH and HHO algorithms. In addition, it is observed that these algorithms have a low dispersion in the data.

P10 Problem (Details in Definition A10): In this problem, the AMH algorithm obtained an optimal value with a mean and standard deviation of 4.44 × 10^16^ ± 0.00. The other algorithms did not demonstrate good performance. These results can be visualised in Figure 17c.

P11 Problem (Details in Definition A11): Figure 17d shows that the algorithms closest to the global optimum are AMH and HHO. In addition, it is observed that these algorithms have a low dispersion in the data. However, considering the results of Table 9, the AMH algorithm is the better performing algorithm with a fitness and a standard deviation of 0.00 ± 0.00. In contrast, the HHO algorithm obtained a fitness and a standard deviation of 1.18 × 10^9^ ± 6.47 × 10^9^.

P12 Problem (Details in Definition A12): In this problem, no algorithm achieved a value lower than the tolerance value 1.00 × 10^−8^. These data can be visually contrasted in Figure 17e. In a complementary manner, the algorithms GA, BAT, FFA, MFO, SCA, JAYA, and DE are not considered in Figure 17e because the total sum of their fitness is greater than 1.00 × 10^10^.

P13 Problem (Details in Definition A13): In this problem, no algorithm achieved a result less than the tolerance value 1.00 × 10^−8^. These data can be contrasted visually in Figure 17f. In a complementary manner, the algorithms GA, BAT, FFA, MVO, MFO, CS, SCA, JAYA, and DE are not considered in Figure 17f because the total sum of their fitness is greater than 1.00 × 10^10^.

#### 3.7.2. Execution Time

**Figure 18:**
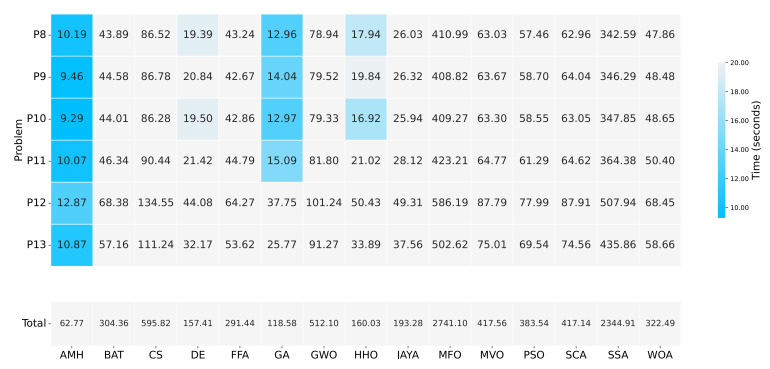
A summary of the execution times of Experiment B. The figure is composed of a matrix and a vector of values that represent a measurement in seconds. The matrix represents the results by a set of cells. The cells indicate the duration of the 31 executions in which each metaheuristic algorithm executed each optimisation problem. The vector represents the total sum for each column of values in the matrix. The calculation is performed by adding together the time values of the problems P8, P9, P10, P11, P12, and P13.

Figure 18 summarises the execution time in which each algorithm solved Experiment B. It can be seen that the AMH algorithm obtained the best time with 62.77 s, the second-best time was obtained by the GA algorithm with 118.58 s, while the DE algorithm obtained the third-best time with 157.41 s.

#### 3.7.3. Ranking

**Figure 19:**
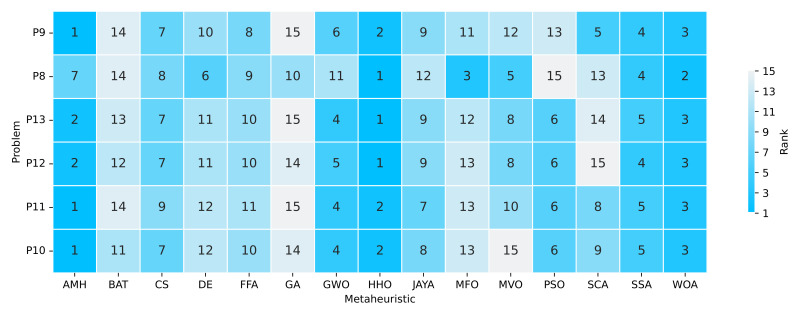
Summary of a ranking matrix between the algorithms in solving optimisation problems, considering mean fitness and execution time indicators. Each row represents the ranking among the 15 algorithms ordered by efficiency in solving a problem P8, P9, P10, P11, P12, and P13.

The ranking determines the position of the algorithm based on the indicators of the best mean fitness values and the shortest execution time. These results are displayed in Figure 19. If we only consider the algorithms that have obtained a ranking of 1, 2 or 3, we can extract the following observations:

- The AMH algorithm obtained three problems at rank 1, two problems at rank 2, and one problem at rank 7.
- The HHO algorithm obtained three problems in rank 1, and three problems in rank 2.
- The WOA algorithm obtained five problems in rank 3, and one in rank 2.
- The MFO algorithm obtained one problem in rank 3.

With this information, we can deduce that the HHO algorithm ranks first place, AMH second place, and WOA third place.

#### 3.7.4. Search Trajectory Networks

The AMH, HHO, and WOA algorithms were chosen as they were the first three algorithms in the ranking described in Figure 19.

**Figure 20:**
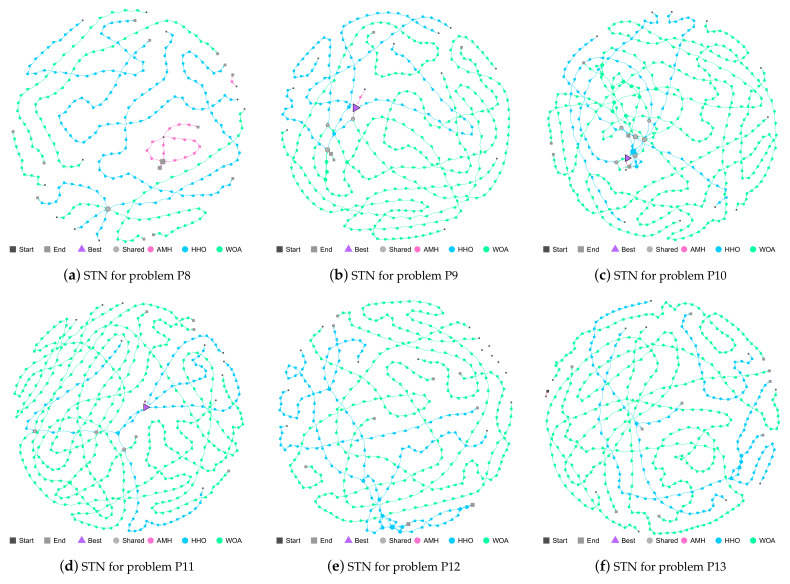
(**a**–**f**) Search Trajectory Networks of the AMH, HHO, and WOA algorithms for problems P8, P9, P10, P11, P12, and P13, respectively. The squares indicate the start and end locations of the algorithm executions. The triangle node is the best-found solution. The circles represent the nodes of algorithms AMH, HHO, and WOA. Each algorithm has a default colour for each circular node. If a circular node is shared by more than one algorithm, it is depicted in light grey.

P8 Problem: No algorithm achieved good fitness results; therefore, the trajectories displayed in Figure 20a do not converge at the best location (triangle node). The HHO and WOA algorithms share a solution (grey circle node). Finally, it can be seen that all the algorithms have explored new solutions.

P9 Problem: In Figure 20b, in general, the five trajectories of the AMH and HHO algorithms have managed to reach the best location (triangle node), ending their location with a mean and standard deviation of 0.00 ± 0.00 for the AMH algorithm and 1.60 × 10^−11^ ± 6.68 × 10^−11^ for the HHO algorithm. There are three solutions shared by the HHO and WOA algorithms (grey circle node).

P10 Problem: In Figure 20c, in general, the trajectories of the AMH and HHO algorithms have managed to reach the best location (triangle node), ending their location with a mean and standard deviation of 0.00 ± 0.00 for the AMH algorithm and 2.97 × 10^−8^ ±6.58 × 10^−8^ for the HHO algorithm. There are six solutions shared by the HHO and WOA algorithms (grey circle node). The WOA algorithm has visually managed to reach close to the best location, this observation can be contrasted by verifying that the mean value and standard deviation bear a value of 1.60 × 10^−2^ ± 3.01 × 10^−2^.

P11 Problem: In Figure 20d, in general, the trajectories of the AMH and HHO algorithms have managed to obtain the best location (triangle node), ending their location with a mean and standard deviation of 0.00 ± 0.00 for the AMH algorithm and 1.18 × 10^−9^ ± 6.47 × 10^−9^ for the HHO algorithm. The algorithm WOA has not reached the best location (triangle node); however, it shares three solutions with the HHO algorithm.

P12 Problem: In this problem, no algorithm achieved a value lower than the tolerance value 1.00 × 10^−8^. The AMH algorithm does not have a trajectory and can be seen in the upper right part of the figure (black square node). The HHO and WOA algorithms have searched the entire search space.

P13 Problem: In this problem, no algorithm achieved value a lower than the tolerance value 1.00 × 10^−8^. The AMH algorithm has a short trajectory. The HHO and WOA algorithms have searched the entire search space.

#### 3.7.5. Convergence

**Figure 21:**
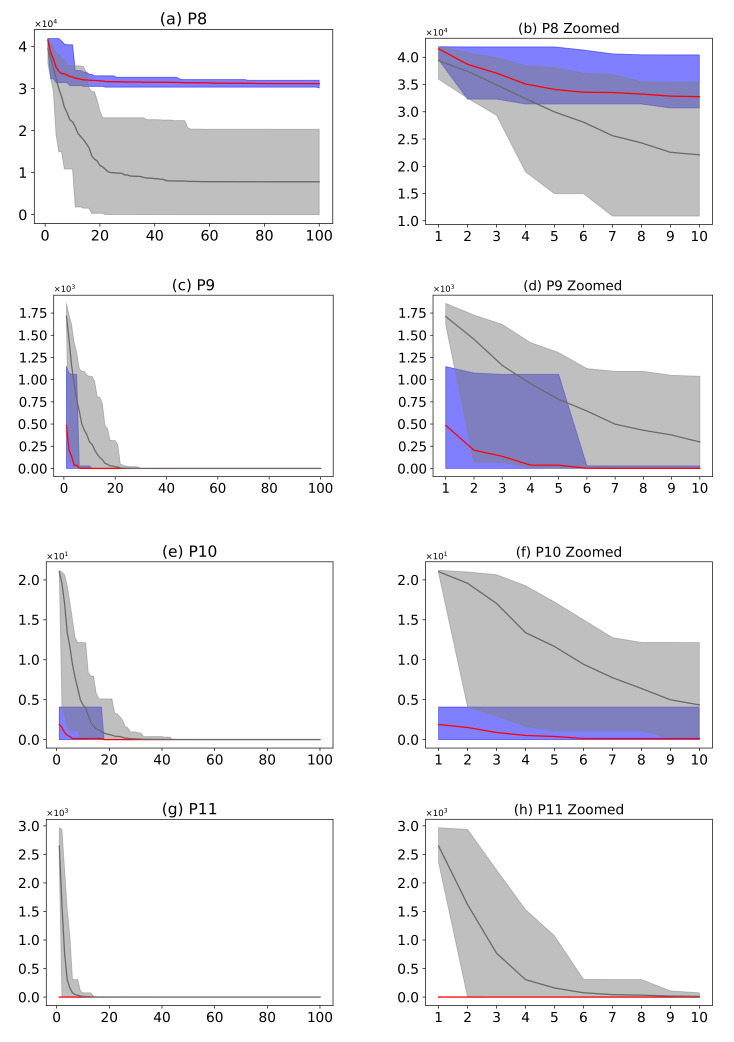

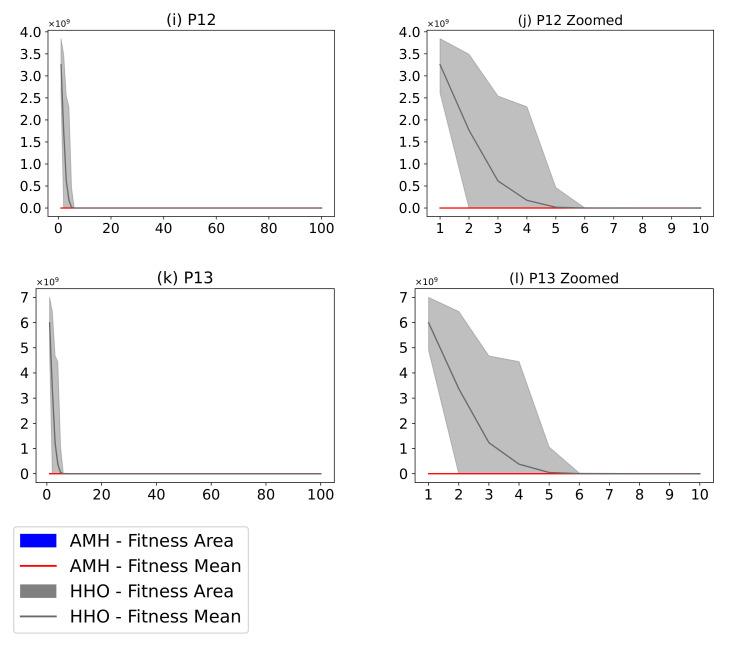
(**Plots a**,**c**,**e**,**g**,**i**,**k**) describe the convergence curves of the AMH and HHO algorithms for problems P1, P2, P3, P4, P5, P6, and P7; (**Plots b**,**d**,**f**,**h**,**j**,**l**) describe an enlarged view of the convergence curve from iteration 1 to 10. The x-axis indicates the number of iterations, and the y-axis indicates fitness. The areas represent the minimum and maximum fitness values obtained in each iteration for each algorithm. The lines represent the mean fitness value of each iteration. Information regarding the 31 executions is included.

Figure 21 describes a comparison between the AMH algorithm and the HHO algorithm. The AMH and HHO algorithms were chosen as they were the first and second ranking algorithms described in Figure 19. Considering the fitness area for problems P9, P10, and P11, based on the mean fitness value, it can be seen in Figure 21c,e,g that under 100 iterations, the AMH algorithm has a faster convergence compared to the HHO algorithm.

Considering the area of fitness for problems P9, P10, and P11, the area of the AMH algorithm tends to be much smaller than the area of fitness of the HHO algorithm. Furthermore, the minimum and maximum values in each iteration of the fitness of the AMH algorithm tend to be smaller in contrast to the minimum and maximum values of the fitness of the HHO algorithm. This observation is also observed between the first 10 iterations in Figure 21d,f,h.

Concerning other observations, in problem P8 of Figure 21a,b, the AMH algorithm loses convergence according to the area. For problems P12 and P13, in Figure 21i–l, it can be seen that the AMH algorithm has a fast convergence compared to the HHO algorithm.

## 4. Discussion

This section describes an overview of the *AutoMH framework* performance, a resume of the comparative experiments, final comments and guidelines for future work of this research.

*Performance*: In the experimental tests, the optimisation problems were considered to have a dimension *D* of 100, which is the maximum dimension described in the competition of the Congress of Evolutionary Computation CEC 2014 [44] and CEC 2015 [45]. In the CEC competition, the optimisation problems tests are conducted with dimensions 10, 30, 50 and 100. In addition, the smallest possible time variable was considered; therefore, as a termination criterion of the algorithms, the maximum number of iterations used in the tests was 100. This number contrasts with the CEC competition in that iterations are calculated with the formula MaxFES=10,000*D, giving 1,000,000 iterations. The restriction of 100 iterations included in this research forces the AutoMH framework to find evolutionary metaheuristic algorithms capable of solving the portfolio of optimisation problems in a stress scenario.

According to the results obtained in experiments A and B, the AMH algorithm generated by the AutoMH framework managed to reach the optimal global value for 9 of the 13 optimisation problems listed as P1, P2, P3, P4, P6, P7, P9, P10, and P11. The results show that the HHO metaheuristic algorithm performed second best, finding the optimal global value for problems P1, P6, P7, P9, and P11. None of the algorithms reached the optimal global value for problems P8, P12, and P13. These results were observed through a ranking perspective choosing the indicators of average fitness and shorter execution time. The AMH algorithm obtained eight problems solved in ranking 1 and four solved in ranking 2. The next best algorithm was the HHO algorithm with five problems in ranking 1 and eight problems in ranking 2, followed by the WOA algorithm with one problem in ranking 2, and 10 problems in ranking 3; finally, the other 12 algorithms tested did not demonstrate noteworthy results. These observations provide supporting evidence that the AMH algorithm generated by the *AutoMH framework* has a performance equal to or better than algorithms reported in the literature.

Considering the Search Trajectory Network graphs, the trajectory of the AMH algorithm is short, with two or three nodes. The trajectory visually tends to be more directed, focusing on solution intensification rather than space exploration, in contrast to the trajectories of the HHO and WOA algorithms that perform more exploration of the search space. This observation can be extended if the convergence of the algorithms is considered for this point. The AMH algorithm visually tends to demonstrate a fast and robust convergence compared to the HHO algorithm.

*Remarks*: This research has fully contributed to the field of machine learning optimisation, specifically in the integration of reinforcement learning for solving optimisation problems. Based on reinforcement learning, the design of the *AutoMH framework* has allowed, through an online evolution process, the automatic generation of viable evolutionary metaheuristic algorithms that are capable of solving a portfolio of optimisation problems posed by the user. The algorithm generated by the *AutoMH framework* has proven to be capable of solving optimisation problems with equal or superior performance compared to the 14 metaheuristic algorithms considered in this study.

*Future Work*: There are several lines to consider for future work, such as integrating new operators or new indivisible functions of intensification and exploration. In such a way, the variety of new metaheuristic algorithms that can be found is enriched. A starting point is to extend the *AutoMH framework* library by considering new number sequences from the On-Line Encyclopedia of Integer Sequences [25]. Another topic is to use a more considerable number of non-intelligent agents to increase the options of having a more significant number of proposed algorithms that solve the set of entered problems and perhaps include new optimisation problems. Finally, another line of research consists of deepening various strategies in the *Action Process* of the *AutoMH framework*. These strategies could focus on methods that generate the ranking of non-intelligent agents in the environment, such as standard competition ranking, modified competition ranking, dense ranking, ordinal ranking, and fractional ranking. Various methods to perform the partition in the *Action Process* additionally warrant further research.

## Figures and Tables

**Table 1:** List of unimodal continuous optimisation problems.

Identifier	Function Name	Domain	$f_{\min}\left(x^{*}\right)$	$x^{*}=\left[x_{1},x_{2},\cdots ,x_{n}\right]$	Details	Reference
P01	Sphere	[−100, 100]	0	f(0, 0, …, 0)	Definition A1	[19,20]
P02	Schwefel Function 2.22	[−10, 10]	0	f(0, 0, …, 0)	Definition A2	[20]
P03	Schwefel Function 1.2	[−100, 100]	0	f(0, 0, …, 0)	Definition A3	[20]
P04	Schwefel Function 2.21	[−100, 100]	0	f(0, 0, …, 0)	Definition A4	[20,21,22]
P05	Rosenbrock’s	[−30, 30]	0	f(1, 1, …, 1)	Definition A5	[19]
P06	Step	[−100, 100]	0	f($x_{1},x_{2},\cdots ,x_{d}$), $x_{i}\in \left[-0.5,0.5\right),\;\;i=\left\{1,2,\ldots ,d\right\}$	Definition A6	[19,20]
P07	Quartic	[−1.28, 1.28]	0	f(0, 0, …, 0)	Definition A7	[20]

A detailed description of each problem can be found in Appendix A.

**Table 2:** List of multimodal continuous optimisation problems.

Identifier	Function Name	Domain	$f_{\min}\left(x^{*}\right)$	$x^{*}=\left[x_{1},x_{2},\ldots ,x_{n}\right]$	Details	Reference
P08	Schwefel Function 2.26	[−500, 500]	0	f(4.21 × 10 ^2^, …, 4.21 × 10 ^2^)	Definition A8	[20]
P09	Rastrigin	[−5.12, 5.12]	0	f(0, 0, … 10 ^2^, 0)	Definition A9	[23]
P10	Ackley	[−32, 32]	0	f(0, 0, …, 0)	Definition A10	[19]
P11	Griewank	[−600, 600]	0	f(0, 0, …, 0)	Definition A11	[24]
P12	Generalized Penalized Function 1	[−50, 50]	0	f(−1, −1, …, −1)	Definition A12	[23]
P13	Generalized Penalized Function 2	[−50, 50]	0	f(1, 1, …, 1)	Definition A13	[23]

A detailed description of each problem can be found in Appendix A.

**Table 3:** List of operators.

Identifier	Name	Math	Code
O00	None	*x* ← *x*	x = x
O01	Plus	*x* ← *x* + *f*(*x*)	x = x + f(x)
O02	Subtract	*x* ← *x* − *f*(*x*)	x = x − f(x)
O03	Multiply	*x* ← *x* * *f*(*x*)	x = x * f(x)
O04	Divide	*x* ← $\frac{x}{f(x)}$	x = x/f(x)

**Table 4:** List of basic functions.

Identifier	Name	Function	Code
I01	Sine	*f*(*x*) = *sin*(*x*)	x = sin(x)
I02	Cosine	*f*(*x*) = *cos*(*x*)	x = cos(x)
I03	Tangent	*f*(*x*) = *tan*(*x*)	x = tan(x)
I04	Inverse Sine	*f*(*x*) = *arcsin*(*x*)	x = arcsin(x)
I05	Inverse Cosine	*f*(*x*) = *arccos*(*x*)	x = arccos(x)
I06	Inverse Tangent	*f*(*x*) = *arctan*(*x*)	x = arctan(x)
I07	Absolute	*f*(*x*) = |*x*|	x = abs(x)
I08	Square root	*f*(*x*) = $\sqrt{x}$	x = sqrt(x)
I09	Exponential function	*f*(*x*) = *e*^*x*^	x = exp(x)
I10	Exponential function minus 1	*f*(*x*) = *e*^*x*^ − 1	x = exp1(x)
I11	Natural logarithm	*f*(*x*) = ln(*x*)	x = ln(x)
I12	Base-2 logarithm of x	*f*(*x*) = log_2_(*x*)	x = log2(x)
I13	Base-10 logarithm of x	*f*(*x*) = log_10_(*x*)	x = log10(x)
I14	Natural logarithm of one plus	*f*(*x*) = ln(1 + *x*)	x = ln(1 + x)

**Table 5:** List of random number functions.

Identifier	Name	Function	Code	Description
I100	Uniform F1	*U*_*f*1_ ~ (*l*, *u*)	x = uniform1(l, u)	
I101	Uniform F2	*U*_*f*2_ ~ (*l*, *u*)	x = uniform2(l, u)	u = lb + (ub − lb)/2
I102	Uniform F3	*U*_*f*3_ ~ (*l*, *u*)	x = uniform3(l, u)	l = lb + (ub − lb)/2
I103	Uniform F4	*U*_*f*4_ ~ (*l*, *u*)	x = uniform4(l, u)	l = lb + (ub − lb)/3 u = lb + (ub − lb)/3*2
I104	Uniform F5	*U*_*f*5_ ~ (*l*, *u*)	x = uniform5(l, u)	u = lb + (ub − lb)/4
I105	Uniform F6	*U*_*f*6_ ~ (*l*, *u*)	x = uniform6(l, u)	l = lb + (ub − lb)/4 u = lb + (ub − lb)/2
I106	Uniform F7	*U*_*f*7_ ~ (*l*, *u*)	x = uniform7(l, u)	l = lb + (ub − lb)/2 u = lb + (ub − lb)/4*3
I107	Uniform F8	*U*_*f*8_ ~ (*l*, *u*)	x = uniform8(l, u)	l = lb + (ub − lb)/4*3
I108	Uniform F9	*U*_*f*9_ ~ (−1, 1)	x = uniform9(−1, 1)	
I109	Uniform F10	*U*_*f*10_ ~ (0, 1)	x = uniform10(0, 1)	
I110	Uniform F11	*U*_*f*11_ ~ (−1, 1)	x = uniform11(−1, 0)	
I111	Uniform F12	*U*_*f*12_ ~ (0.5, 0.5)	x = uniform12(0.5, 0.5)	
I112	Beta F1	*B*_*f*1_ ~ (0.5, 0.5, 1)	x = beta1(0.5, 0.5, 1)	
I113	Beta F2	*B*_*f*2_ ~ (5, 1, 1)	x = beta2(5, 1, 1)	
I114	Beta F3	*B*_*f*3_ ~ (1, 3, 1)	x = beta3(1, 3, 1)	
I115	Beta F4	*B*_*f*4_ ~ (2, 2, 1)	x = beta4(2, 2, 1)	
I116	Beta F5	*B*_*f*5_ ~ (2, 5, 1)	x = beta5(2, 5, 1)	
I117	Triangular F1	*T*_*f*1_ ~ (*lb*, *m*, *ub*)	x = triangular1(lb, m, ub)	m = lb + (ub − lb)/2
I118	Triangular F2	*T*_*f*2_ ~ (*lb*, *m*, *ub*)	x = triangular2(lb, m, ub)	m = lb + (ub − lb)/4
I119	Triangular F3	*T*_*f*3_ ~ (*lb*, *m*, *ub*)	x = triangular3(lb, m, ub)	m = lb + (ub − lb)/3
I120	Triangular F4	*T*_*f*4_ ~ (*lb*, *m*, *ub*)	x = triangular4(lb, m, ub)	m = lb + ((ub − lb)/4)*3
I121	Triangular F5	*T*_*f*5_ ~ (*lb*, *m*, *ub*)	x = triangular5(lb, m, ub)	m = lb + ((ub − lb)/3)*2

**Table 6:** List of constants.

ID	Name	Symbol	Value	Code
I200	Meissel–Mertens	$M_{1}$	0.26149 72128 47642 78375 54268 38608 69585	A077761( )
I201	Bernstein’s	β	0.28016 94990 23869 13303	A073001( )
I202	Gauss–Kuzmin–Wirsing	λ	0.30366 30028 98732 65859 74481 21901 55623	A038517( )
I203	Hafner–Sarnak–McCurley	σ	0.35323 63718 54995 98454 35165 50432 68201	A085849( )
I204	Omega	Ω	0.56714 32904 09783 87299 99686 62210 35554	A030178( )
I205	Euler–Mascheroni	γ	0.57721 56649 01532 86060 65120 90082 40243	A001620( )
I206	Twin prime	$C_{2}$	0.66016 18158 46869 57392 78121 10014 55577	A005597( )
I207	Conway’s	$\lambda_{c}$	1.30357 72690 34296 39125 70991 12152 55189	A014715( )
I208	Ramanujan–Soldner	μ	1.45136 92348 83381 05028 39684 85892 02744	A070769( )
I209	Golden ratio	φ	1.61803 39887 49894 84820 45868 34365 63811	A001622( )
I210	Euler’s number	*e*	2.71828 18284 59045 23536 02874 71352 66249	A001113( )
I211	Pi	π	3.14159 26535 89793 23846 26433 83279 50288	A000796( )
I212	Reciprocal Fibonacci	ψ	3.35988 56662 43177 55317 20113 02918 92717	A079586( )

**Table 7:** Parameters used in the AutoMH experiment.

ID	Name	Description	Value
T01	Evolutionary Agents	The number of non-intelligent agents in the swarm.	agents=10
T02	Evolutionary Iterations (Episode)	The number of times the agents in the swarm have to repeat the optimisation tests once the structure of their algorithm is modified by the *learning agent*.	episodes=100
T03	Mutation Selection	The mutation is carried out by randomly choosing an action of the type Add, Replace, and Remove for the Modified case.	mutation=random
T04	MH Iteration	The maximum number of iterations that the metaheuristic executes.	iterations=100
T05	MH Execution	The number of times the metaheuristic is executed.	executions=31
T05	MH Probability	The probability of choosing intensification or exploration in the Step function.	P=0.5
T06	Dimension	The dimension of optimisation problems.	D=100
T07	Operator Initial	The operators Δ allowed to modify the metaheuristic template in the Initial function.	Δ={O01,O02,O03,O04}
T08	Initial Functions	The Initial functions h(x) allowed for modifying the metaheuristic template in the Initial function.	$\begin{array}{l} h(x)=\{I100,I101,I102,I103,I104,I105,\\ I106,I107,I108,I109,I110,I111,I112,I113,\\ I114,I120,I123,I130,I131,I132,I133,I134,\\ I200,I201,I202,I203,I204,I205,I206,I207,\\ I208,I209,I210,I211,I212\} \end{array}$
T09	Operator Exploration	The operators Δ allowed to modify the metaheuristic template in the step function.	Δ={O01,O02,O03,O04}
T10	Exploration Functions	The exploration instructions g(x) allowed for modifying the metaheuristic template in the step function.	$\begin{array}{l} g(x)=\{I100,I101,I102,I103,I104,I105,\\ I106,I107,I108,I109,I110,I111,I112,I113,\\ I114,I120,I123,I130,I131,I132,I133,I134,\\ I200,I201,I202,I203,I204,I205,I206,I207,\\ I208,I209,I210,I211,I212\} \end{array}$
T11	Operator Intensification	The operators Δ allowed to modify the metaheuristic template in the step function.	Δ={O01,O02,O03,O04}
T12	Intensification Functions	The intensification functions h(x) allowed for the modification of the metaheuristic template in the step function.	$\begin{array}{l} h(x)=\{I01,I02,I03,I04,I05,I06,I07,I08,\\ I09,I10,I11,I12,I13,I14\} \end{array}$
T13	Initial quantity	Minimum and maximum number of operators allowed in the generated metaheuristic	$min_{value}=1\;\;\;max_{value}=5$.
T14	Exploration quantity	Minimum and maximum amount of exploration instructions allowed in the generated metaheuristic	$min_{value}=1\;\;\;max_{value}=10$.
T15	Intensification quantity	Minimum and maximum amount of intensification instructions allowed in the generated metaheuristic	$min_{value}=1\;\;\;max_{value}=10$.

**Table 8:** Experiment A: Statistical Summary.

Metaheuristic	Type	P1	P2	P3	P4	P5	P6	P7
AMH	mean	**0.00**	**0.00**	**0.00**	**0.00**	9.90 × 10${}^{1}$	**0.00**	**0.00**
	std	0.00	0.00	0.00	0.00	0.00	0.00	0.00
BAT	mean	1.74 × 10${}^{5}$	1.54 × 10${}^{47}$	2.16 × 10${}^{6}$	8.67 × 10${}^{1}$	5.51 × 10${}^{8}$	1.80 × 10${}^{5}$	7.56 × 10${}^{2}$
	std	6.19 × 10${}^{4}$	7.53 × 10${}^{47}$	1.43 × 10${}^{6}$	1.02 × 10${}^{1}$	3.27 × 10${}^{8}$	7.11 × 10${}^{4}$	4.60 × 10${}^{2}$
CS	mean	6.98 × 10${}^{4}$	1.65 × 10${}^{14}$	1.79 × 10${}^{5}$	6.46 × 10${}^{1}$	1.13 × 10${}^{8}$	6.98 × 10${}^{4}$	1.73 × 10${}^{2}$
	std	1.18 × 10${}^{4}$	8.53 × 10${}^{14}$	5.26 × 10${}^{4}$	6.19	3.21 × 10${}^{7}$	1.20 × 10${}^{4}$	5.06 × 10${}^{1}$
DE	mean	1.64 × 10${}^{5}$	4.39 × 10${}^{21}$	4.11 × 10${}^{5}$	8.57 × 10${}^{1}$	5.48 × 10${}^{8}$	1.65 × 10${}^{5}$	9.00 × 10${}^{2}$
	std	2.56 × 10${}^{4}$	2.21 × 10${}^{22}$	1.72 × 10${}^{5}$	6.55	1.73 × 10${}^{8}$	2.64 × 10${}^{4}$	2.49 × 10${}^{2}$
FFA	mean	1.19 × 10${}^{5}$	4.06 × 10${}^{26}$	3.80 × 10${}^{5}$	8.59 × 10${}^{1}$	2.72 × 10${}^{8}$	1.18 × 10${}^{5}$	3.63 × 10${}^{2}$
	std	1.71 × 10${}^{4}$	2.20 × 10${}^{27}$	9.36 × 10${}^{4}$	4.49	6.18 × 10${}^{7}$	1.59 × 10${}^{4}$	1.15 × 10${}^{2}$
GA	mean	2.04 × 10${}^{5}$	1.04 × 10${}^{37}$	5.52 × 10${}^{5}$	9.25 × 10${}^{1}$	7.43 × 10${}^{8}$	2.02 × 10${}^{5}$	1.18 × 10${}^{3}$
	std	1.61 × 10${}^{4}$	3.78 × 10${}^{37}$	1.45 × 10${}^{5}$	1.84	9.78 × 10${}^{7}$	1.32 × 10${}^{4}$	1.62 × 10${}^{2}$
GWO	mean	2.27 × 10${}^{3}$	2.60 × 10${}^{1}$	1.29 × 10${}^{5}$	5.55 × 10${}^{1}$	1.31 × 10${}^{6}$	2.45 × 10${}^{3}$	1.25
	std	5.16 × 10${}^{2}$	4.58	2.59 × 10${}^{4}$	5.68	7.54 × 10${}^{5}$	7.15 × 10${}^{2}$	8.56 × 10${}^{-1}$
HHO	mean	$1.96\times 10^{-11}$	2.16 × 10${}^{-6}$	7.94 × 10${}^{2}$	1.99 × 10${}^{-6}$	1.12 × 10${}^{1}$	**0.00**	$1.65\times 10^{-28}$
	std	1.03 × 10${}^{-10}$	8.77 × 10${}^{-6}$	4.35 × 10${}^{3}$	7.53 × 10${}^{-6}$	2.41 × 10${}^{1}$	0.00	7.75 × 10${}^{-28}$
JAYA	mean	5.60 × 10${}^{4}$	1.81 × 10${}^{2}$	5.53 × 10${}^{5}$	9.60 × 10${}^{1}$	1.84 × 10${}^{8}$	5.37 × 10${}^{4}$	2.58 × 10${}^{2}$
	std	1.45 × 10${}^{4}$	5.05 × 10${}^{1}$	1.37 × 10${}^{5}$	2.58	5.24 × 10${}^{7}$	1.50 × 10${}^{4}$	7.45 × 10${}^{1}$
MFO	mean	1.67 × 10${}^{5}$	7.86 × 10${}^{16}$	3.80 × 10${}^{5}$	9.22 × 10${}^{1}$	5.85 × 10${}^{8}$	1.67 × 10${}^{5}$	8.41 × 10${}^{2}$
	std	1.57 × 10${}^{4}$	4.30 × 10${}^{17}$	8.00 × 10${}^{4}$	1.87	9.61 × 10${}^{7}$	1.45 × 10${}^{4}$	1.20 × 10${}^{2}$
MVO	mean	8.40 × 10${}^{4}$	9.47 × 10${}^{36}$	2.70 × 10${}^{5}$	8.78 × 10${}^{1}$	1.85 × 10${}^{8}$	8.18 × 10${}^{4}$	2.54 × 10${}^{2}$
	std	1.17 × 10${}^{4}$	3.42 × 10${}^{37}$	4.96 × 10${}^{4}$	3.51	5.35 × 10${}^{7}$	1.21 × 10${}^{4}$	8.73 × 10${}^{1}$
PSO	mean	2.16 × 10${}^{4}$	9.87 × 10${}^{18}$	1.46 × 10${}^{5}$	4.14 × 10${}^{1}$	2.04 × 10${}^{7}$	1.98 × 10${}^{4}$	1.38 × 10${}^{3}$
	std	5.94 × 10${}^{3}$	5.41 × 10${}^{19}$	5.32 × 10${}^{4}$	4.12	1.97 × 10${}^{7}$	3.99 × 10${}^{3}$	2.52 × 10${}^{3}$
SCA	mean	5.81 × 10${}^{4}$	6.49 × 10${}^{1}$	5.84 × 10${}^{5}$	9.68 × 10${}^{1}$	5.46 × 10${}^{8}$	5.80 × 10${}^{4}$	7.76 × 10${}^{2}$
	std	2.62 × 10${}^{4}$	2.97 × 10${}^{1}$	2.06 × 10${}^{5}$	1.37	1.94 × 10${}^{8}$	2.58 × 10${}^{4}$	3.11 × 10${}^{2}$
SSA	mean	1.07 × 10${}^{4}$	8.05 × 10${}^{1}$	4.93 × 10${}^{4}$	2.57 × 10${}^{1}$	2.62 × 10${}^{6}$	1.11 × 10${}^{4}$	3.85
	std	1.45 × 10${}^{3}$	7.33	2.43 × 10${}^{4}$	2.33	8.43 × 10${}^{5}$	1.52 × 10${}^{3}$	1.15
WOA	mean	8.15 × 10${}^{-2}$	7.91 × 10${}^{-3}$	2.21 × 10${}^{6}$	8.23 × 10${}^{1}$	7.14 × 10${}^{2}$	1.58	1.49 × 10${}^{-3}$
	std	1.74 × 10${}^{-1}$	3.68 × 10${}^{-2}$	1.49 × 10${}^{6}$	1.84 × 10${}^{1}$	2.62 × 10${}^{3}$	2.64	7.43 × 10${}^{-3}$

If the mean value of the fitness is less than the tolerance value 1.00 × 10^−8^, these values will be marked in bold.

**Table 9:** Experiment B: Statistical Summary.

Metaheuristic	Type	P8	P9	P10	P11	P12	P13
AMH	mean	$3.12\times 10^{4}$	**0.00**	$4.44\times 10^{-16}$	**0.00**	2.31 × 10^−1^	1.00 × 10 ^1^
	std	4.39 × 10${}^{2}$	0.00	0.00	0.00	5.47 × 10^−2^	0.00
BAT	mean	3.73 × 10${}^{4}$	1.39 × 10${}^{3}$	1.98 × 10${}^{2}$	1.57 × 10${}^{3}$	1.20 × 10${}^{9}$	2.44 × 10${}^{9}$
	std	1.74 × 10${}^{3}$	1.46 × 10${}^{2}$	3.23 × 10${}^{-1}$	5.59 × 10${}^{2}$	9.25 × 10${}^{8}$	1.60 × 10${}^{9}$
CS	mean	3.15 × 10${}^{4}$	9.75 × 10${}^{2}$	1.80 × 10${}^{1}$	6.29 × 10${}^{2}$	1.20 × 10${}^{8}$	3.43 × 10${}^{8}$
	std	6.87 × 10${}^{2}$	4.50 × 10${}^{1}$	6.16 × 10${}^{-1}$	1.06 × 10${}^{2}$	6.18 × 10${}^{7}$	1.33 × 10${}^{8}$
DE	mean	3.03 × 10${}^{4}$	1.12 × 10${}^{3}$	1.99 × 10${}^{1}$	1.48 × 10${}^{3}$	1.14 × 10${}^{9}$	2.36 × 10${}^{9}$
	std	1.57 × 10${}^{3}$	9.65 × 10${}^{1}$	3.07 × 10${}^{-1}$	2.30 × 10${}^{2}$	5.17 × 10${}^{8}$	8.72 × 10${}^{8}$
FFA	mean	3.30 × 10${}^{4}$	1.06 × 10${}^{3}$	1.94 × 10${}^{1}$	1.07 × 10${}^{3}$	4.04 × 10${}^{8}$	9.92 × 10${}^{8}$
	std	1.81 × 10${}^{3}$	7.41 × 10${}^{1}$	2.94 × 10${}^{-1}$	1.54 × 10${}^{2}$	1.40 × 10${}^{8}$	3.03 × 10${}^{8}$
GA	mean	3.30 × 10${}^{4}$	1.39 × 10${}^{3}$	2.06 × 10${}^{1}$	1.84 × 10${}^{3}$	1.53 × 10${}^{9}$	3.04 × 10${}^{9}$
	std	1.42 × 10${}^{3}$	4.60 × 10${}^{1}$	1.23 × 10${}^{-1}$	1.44 × 10${}^{2}$	2.53 × 10${}^{8}$	4.04 × 10${}^{8}$
GWO	mean	3.35 × 10${}^{4}$	6.98 × 10${}^{2}$	7.04	2.14 × 10${}^{1}$	1.05 × 10${}^{5}$	9.72 × 10${}^{5}$
	std	2.94 × 10${}^{3}$	1.15 × 10${}^{2}$	8.72 × 10${}^{-1}$	4.65	2.32 × 10${}^{5}$	1.05 × 10${}^{6}$
HHO	mean	7.82 × 10${}^{3}$	$1.60\times 10^{-11}$	2.97 × 10${}^{-8}$	$1.18\times 10^{-9}$	2.58 × 10${}^{-3}$	1.34 × 10${}^{-1}$
	std	6.32 × 10${}^{3}$	6.68 × 10${}^{-11}$	6.58 × 10${}^{-8}$	6.47 × 10${}^{-9}$	3.73 × 10${}^{-3}$	2.17 × 10${}^{-1}$
JAYA	mean	3.38 × 10${}^{4}$	1.06 × 10${}^{3}$	1.81 × 10${}^{1}$	5.05 × 10${}^{2}$	3.60 × 10${}^{8}$	6.95 × 10${}^{8}$
	std	1.34 × 10${}^{3}$	1.08 × 10${}^{2}$	9.71 × 10${}^{-1}$	1.30 × 10${}^{2}$	1.61 × 10${}^{8}$	2.23 × 10${}^{8}$
MFO	mean	2.56 × 10${}^{4}$	1.17 × 10${}^{3}$	2.02 × 10${}^{1}$	1.50 × 10${}^{3}$	1.25 × 10${}^{9}$	2.44 × 10${}^{9}$
	std	1.32 × 10${}^{3}$	6.57 × 10${}^{1}$	9.02 × 10${}^{-2}$	1.41 × 10${}^{2}$	2.32 × 10${}^{8}$	4.66 × 10${}^{8}$
MVO	mean	2.83 × 10${}^{4}$	1.26 × 10${}^{3}$	2.06 × 10${}^{1}$	7.57 × 10${}^{2}$	2.81 × 10${}^{8}$	6.41 × 10${}^{8}$
	std	1.29 × 10${}^{3}$	6.05 × 10${}^{1}$	2.03 × 10${}^{-1}$	1.09 × 10${}^{2}$	9.98 × 10${}^{7}$	2.32 × 10${}^{8}$
PSO	mean	3.75 × 10${}^{4}$	1.37 × 10${}^{3}$	1.50 × 10${}^{1}$	4.57 × 10${}^{2}$	1.23 × 10${}^{6}$	1.17 × 10${}^{7}$
	std	9.52 × 10${}^{2}$	7.13 × 10${}^{1}$	1.33	8.26 × 10${}^{1}$	1.19 × 10${}^{6}$	1.06 × 10${}^{7}$
SCA	mean	3.67 × 10${}^{4}$	5.56 × 10${}^{2}$	1.92 × 10${}^{1}$	5.24 × 10${}^{2}$	1.63 × 10${}^{9}$	2.52 × 10${}^{9}$
	std	5.64 × 10${}^{2}$	2.26 × 10${}^{2}$	2.28	2.36 × 10${}^{2}$	4.14 × 10${}^{8}$	8.83 × 10${}^{8}$
SSA	mean	2.76 × 10${}^{4}$	5.39 × 10${}^{2}$	1.17 × 10${}^{1}$	9.70 × 10${}^{1}$	4.86 × 10${}^{3}$	1.27 × 10${}^{6}$
	std	1.34 × 10${}^{3}$	3.33 × 10${}^{1}$	7.38 × 10${}^{-1}$	1.30 × 10${}^{1}$	9.64 × 10${}^{3}$	7.12 × 10${}^{5}$
WOA	mean	1.47 × 10${}^{4}$	7.30	1.60 × 10${}^{-2}$	1.56 × 10${}^{-1}$	2.11 × 10${}^{1}$	1.53 × 10${}^{2}$
	std	5.29 × 10${}^{3}$	3.57 × 10${}^{1}$	3.01 × 10${}^{-2}$	2.85 × 10${}^{-1}$	6.84 × 10${}^{1}$	4.38 × 10${}^{2}$

If the mean value of the fitness is less than the tolerance value 1.00 × 10^−8^, these values will be marked in bold.

## Data Availability

https://doi.org/10.6084/m9.figshare.19469579, accessed on 31 March 2022.

## Abbreviations

The following abbreviations are used in this manuscript:

BAT	Bat Algorithm
CS	Cuckoo Search
DE	Differential Evolution
FFA	FireFly Algorithm
GA	Genetic Algorithm
GWO	Grey Wolf Optimiser
HHO	Harris Hawks Optimization
JAYA	Jaya algorithm
MFO	Moth-Flame optimisation
MVO	Multi-Verse Optimiser
PSO	Particle Swarm Optimisation
SCA	Sine Cosine optimisation Algorithm
SSA	Salp Swarm Algorithm
WOA	Whale Optimization Algorithm

## Appendix A. Benchmark

The appendix contains details of the continuous optimisation dataset for P01 to P13 functions. Problems P01 to P07 correspond to unimodal functions, and problems P08 to P13 correspond to multimodal functions.

**Definition** **A1.**
*P01—Sphere. The Sphere function is defined by the objective function (A1). The function is defined and evaluated in the input domain $x_{i}\in \left[-100,100\right]$ for all i={1,2,…,d}. The function has one global minimum at $f_{min}\left(x^{*}\right)=0$ with $x^{*}=\left[0,0,\ldots ,0\right]$.*

(A1)
$$\begin{array}{c} f\left(x\right)=\sum_{i=1}^{d}x_{i}^{2} \end{array}$$

**Definition** **A2.**
*P02—Schwefel 2.22. The Schwefel 2.22 function is defined by the objective function (A2). The function is defined and evaluated in the input domain $x_{i}\in \left[-10,10\right]$ for all i={1,2,…,d}. The function has one global minimum at $f_{min}\left(x^{*}\right)=0$ with $x^{*}=\left[0,0,\ldots ,0\right]$.*

(A2)
$$\begin{array}{c} f\left(x\right)=\sum_{i=1}^{d}|x_{i}|+\prod_{i=1}^{d}\left|x_{i}\right| \end{array}$$

**Definition** **A3.**
*P03—Schwefel 1.2. The Schwefel 1.2 function is defined by the objective function (A3). The function is defined and evaluated in the input domain $x_{i}\in \left[-100,100\right]$ for all i={1,2,…,d}. The function has one global minimum at $f_{min}\left(x^{*}\right)=0$ with $x^{*}=\left[0,0,\ldots ,0\right]$.*

(A3)
$$\begin{array}{c} f\left(x\right)=\sum_{i=1}^{d}{\left(\sum_{j=1}^{i}x_{j}\right)}^{2} \end{array}$$

**Definition** **A4.**
*P04—Schwefel 2.21. The Schwefel 2.21 function is defined by the objective function (A4). The function is defined and evaluated in the input domain $x_{i}\in \left[-100,100\right]$ for all i={1,2,⋯,d}. The function has one global minimum at $f_{min}\left(x^{*}\right)=0$ with $x^{*}=\left[0,0,\ldots ,0\right]$.*

(A4)
$$\begin{array}{c} f\left(x\right)=\max_{i=1,\ldots ,d}\left|x_{i}\right| \end{array}$$

**Definition** **A5.**
*P05—Rosenbrock’s. The Rosenbrock’s function is defined by the objective function (A5). The function is defined and evaluated in the input domain $x_{i}\in \left[-30,30\right]$ for all i={1,2,…,d}. The function has one global minimum at $f_{min}\left(x^{*}\right)=0$ with $x^{*}=\left[1,1,\ldots ,1\right]$.*

(A5)
$$\begin{array}{c} f\left(x\right)=\sum_{i=1}^{d-1}\left[100{\left(x_{i+1}-x_{i}^{2}\right)}^{2}+{\left(1-x_{i}\right)}^{2}\right] \end{array}$$

**Definition** **A6.**
*P06—Step. The Step function is defined by the objective function (A6). The function is defined and evaluated in the input domain $x_{i}\in \left[-100,100\right]$ for all i={1,2,…,d}. The function has one global minimum at $f_{min}\left(x^{*}\right)=0$ with $x^{*}=\left[x_{1},x_{2},\ldots ,x_{d}\right],x_{i}\in \left[-0.5,0.5\right)$.*

(A6)
$$\begin{array}{c} f\left(x\right)=\sum_{i=1}^{d}{\left(\left\lfloor x_{i}+0.5\right\rfloor \right)}^{2} \end{array}$$

**Definition** **A7.**
*P07—Noisy Quartic. The Noisy Quartic function is defined by the objective function (A7). The function is defined and evaluated in the input domain $x_{i}\in \left[-1.28,1.28\right]$ for all i={1,2,…,d}. The function has one global minimum at $f_{min}\left(x^{*}\right)=0+\sum_{i=1}^{d}\eta_{i}$ with $x^{*}=\left[0,0,\ldots ,0\right]$. Where, η is a random number bounded between [0,1).*

(A7)
$$\begin{array}{c} f\left(x\right)=\sum_{i=1}^{d}\left(ix_{i}^{4}+\eta_{i}\right) \end{array}$$

**Definition** **A8.**
*P08—Schwefel Function 2.26. The Schwefel function 2.26 is defined by the objective function (A8). The function is defined and evaluated in the input domain $x_{i}\in \left[-500,500\right]$ for all i={1,2,…,d}. The function has one global minimum at $f_{min}\left(x^{*}\right)=0$ with $x^{*}=\left[4.209687462275036e+002,4.209687462275036e+002,\ldots ,4.209687462275036e+002\right]$.*

(A8)
$$\begin{array}{c} f\left(x\right)=4.189828872724338e+002\times d-\sum_{i=1}^{d}x_{i}sin\left(\sqrt{|x_{i}|}\right) \end{array}$$

**Definition** **A9.**
*P09—Rastrigin Function. The Rastrigin function 2.26 is defined by the objective function (A9). The function is defined and evaluated in the input domain $x_{i}\in \left[-5.12,5.12\right]$ for all i={1,2,…,d}. The function has one global minimum at $f_{min}\left(x^{*}\right)=0$ with $x^{*}=\left[0,0,\ldots ,0\right]$.*

(A9)
$$\begin{array}{c} f\left(x\right)=\sum_{i=1}^{d}\left[x_{i}^{2}+10\left(1-cos\left(2\pi x_{i}\right)\right)\right] \end{array}$$

**Definition** **A10.**
*P10—Ackley Function. The Ackley function is defined by the objective function (A10). The function is defined and evaluated in the input domain $x_{i}\in \left[-32,32\right]$ for all i={1,2,…,d}. The function has one global minimum at $f_{min}\left(x^{*}\right)=0$ with $x^{*}=\left[0,0,\ldots ,0\right]$.*

(A10)
$$\begin{array}{cc} f(x) & =-a\;\;exp\left(-b\sqrt{\frac{1}{d}\sum_{i=1}^{d}x_{i}^{2}}\right)-exp\left(\frac{1}{d}\sum_{i=1}^{d}cos\left(cx_{i}\right)\right)+a+exp\left(1\right)\\ a & =20\\ b & =0.2\\ c & =2\pi \end{array}$$

**Definition** **A11.**
*P11—Griewank function. The Griewank function is defined by the objective function (A11). The function is defined and evaluated in the input domain $x_{i}\in \left[-600,600\right]$ for all i={1,2,…,d}. The function has one global minimum at $f_{min}\left(x^{*}\right)=0$ with $x^{*}=\left[0,0,\ldots ,0\right]$.*

(A11)
$$\begin{array}{c} f\left(x\right)=\frac{1}{4000}\sum_{i=1}^{d}x_{i}^{2}-\prod_{i=1}^{d}\cos\left(\frac{x_{i}}{\sqrt{i}}\right)+1 \end{array}$$

**Definition** **A12.**
*P12—Generalized Penalized Function 1. The Generalized Penalized function 1 is defined by the objective function (A12). The function is defined and evaluated in the input domain $x_{i}\in \left[-50,50\right]$ for all i={1,2,…,d}. The function has one global minimum at $f_{min}\left(x^{*}\right)=0$ with $x^{*}=\left[-1,-1,\ldots ,-1\right]$.*

(A12)
$$\begin{array}{cc} f(x) & =\frac{\pi }{d}\times \left\{10\sin^{2}\left(\pi y_{1}\right)+\sum_{i=1}^{d-1}\left[{\left(y_{i}-1\right)}^{2}\left(1+10\sin^{2}\left(\pi y_{i+1}\right)\right)\right]+{\left(y_{d}-1\right)}^{2}\right\}+\\ \; & \;\;\;\;\sum_{i=1}^{d}u\left(x_{i},a,k,m\right)\\ y_{i} & =1+\frac{1}{4}\left(x_{i}+1\right)\;\;\;\;\;\;u\left(x_{i},a,k,m\right)=\left\{\begin{array}{cc} k{\left(x_{i}-a\right)}^{m} & \;if\;x_{i}>a\\ 0 & \;if\;-a\leq x_{i}\leq a\\ k{\left(-x_{i}-a\right)}^{m} & \;if\;x_{i}<-a \end{array}\right.\\ a & =10\\ k & =100\\ \;\;m & =4 \end{array}$$

**Definition** **A13.**
*P13—Generalized Penalized Function 2. The Generalized Penalized function 2 is defined by the objective function (A13). The function is defined and evaluated in the input domain $x_{i}\in \left[-50,50\right]$ for all i={1,2,…,d}. The function has one global minimum at $f_{min}\left(x^{*}\right)=0$ with $x^{*}=\left[1,1,\ldots ,1\right]$.*

(A13)
$$\begin{array}{cc} f(x) & =0.1\times \{\sin^{2}\left(3\pi x_{1}\right)+\sum_{i=1}^{d-1}\left[{\left(x_{i}-1\right)}^{2}\left(1+\sin^{2}\left(3\pi x_{i+1}\right)\right)\right]+\\ \; & \;\;\;\;\left[{\left(x_{n}-1\right)}^{2}\left(1+\sin^{2}\left(2\pi x_{n}\right)\right)\right]\}+\sum_{i=1}^{d}u\left(x_{i},a,k,m\right)\\ y_{i} & =1+\frac{1}{4}\left(x_{i}+1\right)\;\;\;\;\;\;u\left(x_{i},a,k,m\right)=\left\{\begin{array}{cc} k{\left(x_{i}-a\right)}^{m} & \;if\;x_{i}>a\\ 0 & \;if\;-a\leq x_{i}\leq a\\ k{\left(-x_{i}-a\right)}^{m} & \;if\;x_{i}<-a \end{array}\right.\\ a & =5\\ k & =100\\ m & =4 \end{array}$$

## Appendix B. Complementary Statistical Test

This section shows complement statistical results demonstrating the significant differences between the AMH, BAT, CS, DE, FFA, GA, GWO, HHO, JAYA, MFO, MVO, PSO, SCA, SSA, and WOA. The results employ the nonparametric multiple test procedure for many-to-one comparisons [46,47]. The results of the *p*-values of the tests are described in Table A1 and Table A2.

The test requires checking for normality of the samples using the Kolmogorov–Smirnov test [48]. The Kolmogorov–Smirnov test conditions are as follows:

- $H_{0}$: Null hypothesis assumes that the population is normally distributed.
- $H_{A}$: Alternative hypothesis assumes that the population is not-normally distributed.
- Reject the null hypothesis $H_{0}$ if p<0.05

The Kolmogorov–Smirnov Test results are described in Table A3 and Table A4.

**Table A1:** Nonparametric multiple test—Experiment A *p*-values.

ID	Comparison	P1	P2	P3	P4	P5	P6	P7
1	F(BAT)-F(AMH)	0.0000	0.0000	0.0000	0.0000	0.0000	0.0000	0.0000
2	F(CS)-F(AMH)	0.0000	0.0000	0.0000	0.0000	0.0000	0.0000	0.0000
3	F(DE)-F(AMH)	0.0000	0.0000	0.0000	0.0000	0.0000	0.0000	0.0000
4	F(FFA)-F(AMH)	0.0000	0.0000	0.0000	0.0000	0.0000	0.0000	0.0000
5	F(GA)-F(AMH)	0.0000	0.0000	0.0000	0.0000	0.0000	0.0000	0.0000
6	F(GWO)-F(AMH)	0.0000	0.0000	0.0000	0.0000	0.0000	0.0000	0.0000
7	F(HHO)-F(AMH)	0.0000	0.0000	0.0000	0.0000	0.0000	NA ^1^	0.0000
8	F(JAYA)-F(AMH)	0.0000	0.0000	0.0000	0.0000	0.0000	0.0000	0.0000
9	F(MFO)-F(AMH)	0.0000	0.0000	0.0000	0.0000	0.0000	0.0000	0.0000
10	F(MVO)-F(AMH)	0.0000	0.0000	0.0000	0.0000	0.0000	0.0000	0.0000
11	F(PSO)-F(AMH)	0.0000	0.0000	0.0000	0.0000	0.0000	0.0000	0.0000
12	F(SCA)-F(AMH)	0.0000	0.0000	0.0000	0.0000	0.0000	0.0000	0.0000
13	F(SSA)-F(AMH)	0.0000	0.0000	0.0000	0.0000	0.0000	0.0000	0.0000
14	F(WOA)-F(AMH)	0.0000	0.0000	0.0000	0.0000	0.0114	0.0002	0.0000

^1^ NA (Not Applicable). All 31 runs of the AMHand HHO algorithms have the same results. The results correspond to a fitness value of 0.00.

**Table A2:** Nonparametric multiple test—Experiment B *p*-values.

ID	Comparison	P8	P9	P10	P11	P12	P13
1	F(BAT)-F(AMH)	0.0000	0.0000	0.0000	0.0000	0.0000	0.0000
2	F(CS)-F(AMH)	0.0560	0.0000	0.0000	0.0000	0.0000	0.0000
3	F(DE)-F(AMH)	0.0499	0.0000	0.0000	0.0000	0.0000	0.0000
4	F(FFA)-F(AMH)	0.0000	0.0000	0.0000	0.0000	0.0000	0.0000
5	F(GA)-F(AMH)	0.0000	0.0000	0.0000	0.0000	0.0000	0.0000
6	F(GWO)-F(AMH)	0.0004	0.0000	0.0000	0.0000	0.0000	0.0000
7	F(HHO)-F(AMH)	0.0000	0.0031	0.0000	0.0000	0.0000	0.0000
8	F(JAYA)-F(AMH)	0.0000	0.0000	0.0000	0.0000	0.0000	0.0000
9	F(MFO)-F(AMH)	0.0000	0.0000	0.0000	0.0000	0.0000	0.0000
10	F(MVO)-F(AMH)	0.0000	0.0000	0.0000	0.0000	0.0000	0.0000
11	F(PSO)-F(AMH)	0.0000	0.0000	0.0000	0.0000	0.0000	0.0000
12	F(SCA)-F(AMH)	0.0000	0.0000	0.0000	0.0000	0.0000	0.0000
13	F(SSA)-F(AMH)	0.0000	0.0000	0.0000	0.0000	0.0000	0.0000
14	F(WOA)-F(AMH)	0.0000	0.0000	0.0000	0.0000	0.0000	0.0387

**Table A3:** Kolmogorov–Smirnov Normality Test for Experiment A.

MH	Problem	w	*p*-Value	$H_{0}$	MH	Problem	w	*p*-Value	$H_{0}$
AMH	P1	5.00 × 10 ^−1^	1.08 × 10 ^−7^	Rejected	JAYA	P1	1.00	0.00	Rejected
	P2	5.00 × 10 ^−1^	1.08 × 10 ^−7^	Rejected		P2	1.00	0.00	Rejected
	P3	5.00 × 10 ^−1^	1.08 × 10 ^−7^	Rejected		P3	1.00	0.00	Rejected
	P4	5.00 × 10 ^−1^	1.08 × 10 ^−7^	Rejected		P4	1.00	0.00	Rejected
	P5	1.00	0.00	Rejected		P5	1.00	0.00	Rejected
	P6	5.00 × 10 ^−1^	1.08 × 10 ^−7^	Rejected		P6	1.00	0.00	Rejected
	P7	5.00 × 10 ^−1^	1.08 × 10 ^−7^	Rejected		P7	1.00	0.00	Rejected
BAT	P1	1.00	0.00	Rejected	MFO	P1	1.00	0.00	Rejected
	P2	1.00	0.00	Rejected		P2	1.00	0.00	Rejected
	P3	1.00	0.00	Rejected		P3	1.00	0.00	Rejected
	P4	1.00	0.00	Rejected		P4	1.00	0.00	Rejected
	P5	1.00	0.00	Rejected		P5	1.00	0.00	Rejected
	P6	1.00	0.00	Rejected		P6	1.00	0.00	Rejected
	P7	1.00	0.00	Rejected		P7	1.00	0.00	Rejected
CS	P1	1.00	0.00	Rejected	MVO	P1	1.00	0.00	Rejected
	P2	1.00	0.00	Rejected		P2	1.00	0.00	Rejected
	P3	1.00	0.00	Rejected		P3	1.00	0.00	Rejected
	P4	1.00	0.00	Rejected		P4	1.00	0.00	Rejected
	P5	1.00	0.00	Rejected		P5	1.00	0.00	Rejected
	P6	1.00	0.00	Rejected		P6	1.00	0.00	Rejected
	P7	1.00	0.00	Rejected		P7	1.00	0.00	Rejected
DE	P1	1.00	0.00	Rejected	PSO	P1	1.00	0.00	Rejected
	P2	1.00	0.00	Rejected		P2	1.00	0.00	Rejected
	P3	1.00	0.00	Rejected		P3	1.00	0.00	Rejected
	P4	1.00	0.00	Rejected		P4	1.00	0.00	Rejected
	P5	1.00	0.00	Rejected		P5	1.00	0.00	Rejected
	P6	1.00	0.00	Rejected		P6	1.00	0.00	Rejected
	P7	1.00	0.00	Rejected		P7	1.00	0.00	Rejected
FFA	P1	1.00	0.00	Rejected	SCA	P1	1.00	0.00	Rejected
	P2	1.00	0.00	Rejected		P2	1.00	0.00	Rejected
	P3	1.00	0.00	Rejected		P3	1.00	0.00	Rejected
	P4	1.00	0.00	Rejected		P4	1.00	0.00	Rejected
	P5	1.00	0.00	Rejected		P5	1.00	0.00	Rejected
	P6	1.00	0.00	Rejected		P6	1.00	0.00	Rejected
	P7	1.00	0.00	Rejected		P7	1.00	0.00	Rejected
GA	P1	1.00	0.00	Rejected	SSA	P1	1.00	0.00	Rejected
	P2	1.00	0.00	Rejected		P2	1.00	0.00	Rejected
	P3	1.00	0.00	Rejected		P3	1.00	0.00	Rejected
	P4	1.00	0.00	Rejected		P4	1.00	0.00	Rejected
	P5	1.00	0.00	Rejected		P5	1.00	0.00	Rejected
	P6	1.00	0.00	Rejected		P6	1.00	0.00	Rejected
	P7	1.00	0.00	Rejected		P7	9.92 × 10 ^−1^	6.67 × 10 ^−66^	Rejected
GWO	P1	1.00	0.00	Rejected	WOA	P1	5.00 × 10 ^−1^	1.08 × 10 ^−7^	Rejected
	P2	1.00	0.00	Rejected		P2	5.00 × 10 ^−1^	1.08 × 10 ^−7^	Rejected
	P3	1.00	0.00	Rejected		P3	1.00	0.00	Rejected
	P4	1.00	0.00	Rejected		P4	1.00	0.00	Rejected
	P5	1.00	0.00	Rejected		P5	1.00	0.00	Rejected
	P6	1.00	0.00	Rejected		P6	5.00 × 10 ^−1^	1.08 × 10 ^−7^	Rejected
	P7	6.55 × 10 ^−1^	1.44 × 10 ^−13^	Rejected		P7	5.00 × 10 ^−1^	1.08 × 10 ^−7^	Rejected
HHO	P1	5.00 × 10 ^−1^	1.08 × 10 ^−7^	Rejected					
	P2	5.00 × 10 ^−1^	1.08 × 10 ^−7^	Rejected					
	P3	5.00 × 10 ^−1^	1.08 × 10 ^−7^	Rejected					
	P4	5.00 × 10 ^−1^	1.08 × 10 ^−7^	Rejected					
	P5	5.11 × 10 ^−1^	4.74 × 10 ^−8^	Rejected					
	P6	5.00 × 10 ^−1^	1.08 × 10 ^−7^	Rejected					
	P7	5.00 × 10 ^−1^	1.08 × 10 ^−7^	Rejected					

**Table A4:** Kolmogorov–Smirnov Normality Test for Experiment B.

MH	Problem	w	*p*-Value	$H_{0}$	MH	Problem	w	*p*-Value	$H_{0}$
AMH	P8	1.00	0.00	Rejected	JAYA	P8	1.00	0.00	Rejected
	P9	5.00 × 10 ^−1^	1.08 × 10 ^−7^	Rejected		P9	1.00	0.00	Rejected
	P10	5.00 × 10 ^−1^	1.08 × 10 ^−7^	Rejected		P10	1.00	0.00	Rejected
	P11	5.00 × 10 ^−1^	1.08 × 10 ^−7^	Rejected		P11	1.00	0.00	Rejected
	P12	5.58 × 10 ^−1^	1.26 × 10 ^−9^	Rejected		P12	1.00	0.00	Rejected
	P13	1.00	0.00	Rejected		P13	1.00	0.00	Rejected
BAT	P8	1.00	0.00	Rejected	MFO	P8	1.00	0.00	Rejected
	P9	1.00	0.00	Rejected		P9	1.00	0.00	Rejected
	P10	1.00	0.00	Rejected		P10	1.00	0.00	Rejected
	P11	1.00	0.00	Rejected		P11	1.00	0.00	Rejected
	P12	1.00	0.00	Rejected		P12	1.00	0.00	Rejected
	P13	1.00	0.00	Rejected		P13	1.00	0.00	Rejected
CS	P8	1.00	0.00	Rejected	MVO	P8	1.00	0.00	Rejected
	P9	1.00	0.00	Rejected		P9	1.00	0.00	Rejected
	P10	1.00	0.00	Rejected		P10	1.00	0.00	Rejected
	P11	1.00	0.00	Rejected		P11	1.00	0.00	Rejected
	P12	1.00	0.00	Rejected		P12	1.00	0.00	Rejected
	P13	1.00	0.00	Rejected		P13	1.00	0.00	Rejected
DE	P8	1.00	0.00	Rejected	PSO	P8	1.00	0.00	Rejected
	P9	1.00	0.00	Rejected		P9	1.00	0.00	Rejected
	P10	1.00	0.00	Rejected		P10	1.00	0.00	Rejected
	P11	1.00	0.00	Rejected		P11	1.00	0.00	Rejected
	P12	1.00	0.00	Rejected		P12	1.00	0.00	Rejected
	P13	1.00	0.00	Rejected		P13	1.00	0.00	Rejected
FFA	P8	1.00	0.00	Rejected	SCA	P8	1.00	0.00	Rejected
	P9	1.00	0.00	Rejected		P9	1.00	0.00	Rejected
	P10	1.00	0.00	Rejected		P10	1.00	0.00	Rejected
	P11	1.00	0.00	Rejected		P11	1.00	0.00	Rejected
	P12	1.00	0.00	Rejected		P12	1.00	0.00	Rejected
	P13	1.00	0.00	Rejected		P13	1.00	0.00	Rejected
GA	P8	1.00	0.00	Rejected	SSA	P8	1.00	0.00	Rejected
	P9	1.00	0.00	Rejected		P9	1.00	0.00	Rejected
	P10	1.00	0.00	Rejected		P10	1.00	0.00	Rejected
	P11	1.00	0.00	Rejected		P11	1.00	0.00	Rejected
	P12	1.00	0.00	Rejected		P12	1.00	0.00	Rejected
	P13	1.00	0.00	Rejected		P13	1.00	0.00	Rejected
GWO	P8	1.00	0.00	Rejected	WOA	P8	1.00	0.00	Rejected
	P9	1.00	0.00	Rejected		P9	5.00 × 10 ^−1^	1.08 × 10 ^−7^	Rejected
	P10	1.00	2.15 × 10 ^−242^	Rejected		P10	5.00 × 10 ^−1^	1.08 × 10 ^−7^	Rejected
	P11	1.00	0.00	Rejected		P11	5.00 × 10 ^−1^	1.08 × 10 ^−7^	Rejected
	P12	1.00	0.00	Rejected		P12	5.77 × 10 ^−1^	2.57 × 10 ^−10^	Rejected
	P13	1.00	0.00	Rejected		P13	1.00	3.07 × 10 ^−205^	Rejected
HHO	P8	9.56 × 10 ^−1^	1.67 × 10 ^−42^	Rejected					
	P9	5.00 × 10 ^−1^	1.08 × 10 ^−7^	Rejected					
	P10	5.00 × 10 ^−1^	1.08 × 10 ^−7^	Rejected					
	P11	5.00 × 10 ^−1^	1.08 × 10 ^−7^	Rejected					
	P12	5.00 × 10 ^−1^	1.08 × 10 ^−7^	Rejected					
	P13	5.01 × 10 ^−1^	1.05 × 10 ^−7^	Rejected					

## References

[1] Tovey C.A. (2018). Nature-Inspired Heuristics: Overview and Critique. Recent Advances in Optimization and Modeling of Contemporary Problems.

[2] Kaelbling L.P., Littman M.L., Moore A.W. (1996). Reinforcement learning: A survey. J. Artif. Intell. Res..

[3] Hein D., Hentschel A., Runkler T.A., Udluft S. (2018). Particle swarm optimization for model predictive control in reinforcement learning environments. Critical Developments and Applications of Swarm Intelligence.

[4] Nazari M., Oroojlooy A., Snyder L., Takác M. (2018). Reinforcement learning for solving the vehicle routing problem. Adv. Neural Inf. Process. Syst..

[5] Sadeg S., Hamdad L., Remache A.R., Karech M.N., Benatchba K., Habbas Z. QBSO-FS: A Reinforcement Learning Based Bee Swarm Optimization Metaheuristic for Feature Selection. Proceedings of the 15th International Work-Conference on Artificial Neural Networks, IWANN 2019.

[6] Hayashi K., Ohsaki M. (2020). Reinforcement learning for optimum design of a plane frame under static loads. Engineering with Computers.

[7] Solozabal R., Ceberio J., Takáč M. (2020). Constrained combinatorial optimization with reinforcement learning. arXiv.

[8] Calvet L., de Armas J., Masip D., Juan A.A. (2017). Learnheuristics: Hybridizing metaheuristics with machine learning for optimization with dynamic inputs. Open Math..

[9] Barrett T., Clements W., Foerster J., Lvovsky A. Exploratory combinatorial optimization with reinforcement learning. Proceedings of the AAAI Conference on Artificial Intelligence.

[10] Kanda J., de Carvalho A., Hruschka E., Soares C., Brazdil P. (2016). Meta-learning to select the best meta-heuristic for the traveling salesman problem: A comparison of meta-features. Neurocomputing.

[11] Yu S., Aleti A., Barca J.C., Song A. Hyper-heuristic online learning for self-assembling swarm robots. Proceedings of the 18th International Conference.

[12] De Santiago Júnior V.A., Özcan E., de Carvalho V.R. (2020). Hyper-Heuristics based on Reinforcement Learning, Balanced Heuristic Selection and Group Decision Acceptance. Appl. Soft Comput..

[13] Wai H.T., Yang Z., Wang Z., Hong M. (2018). Multi-agent reinforcement learning via double averaging primal-dual optimization. Adv. Neural Inf. Process. Syst..

[14] Cadenas J.M., Garrido M.C., Muñoz E. (2009). Using machine learning in a cooperative hybrid parallel strategy of metaheuristics. Inf. Sci..

[15] Real E., Liang C., So D., Le Q. AutoML-zero: Evolving machine learning algorithms from scratch. Proceedings of the International Conference on Machine Learning.

[16] Talbi E.G. (2016). Combining metaheuristics with mathematical programming, constraint programming and machine learning. Ann. Oper. Res..

[17] Talbi E.G. (2021). Machine learning into metaheuristics: A survey and taxonomy. ACM Comput. Surv. (CSUR).

[18] Bengio Y., Lodi A., Prouvost A. (2020). Machine learning for combinatorial optimization: A methodological tour d’horizon. Eur. J. Oper. Res..

[19] Back T. (1996). Evolutionary Algorithms in Theory and Practice: Evolution Strategies, Evolutionary Programming, Genetic Algorithms.

[20] Jamil M., Yang X.S. (2013). A literature survey of benchmark functions for global optimisation problems. Int. J. Math. Model. Numer. Optim..

[21] Rosenbrock H. (1960). An automatic method for finding the greatest or least value of a function. Comput. J..

[22] Storn R., Price K. (1997). Differential evolution—A simple and efficient heuristic for global optimization over continuous spaces. J. Glob. Optim..

[23] Ma H., Simon D. (2017). Evolutionary Computation with Biogeography-Based Optimization.

[24] Griewank A.O. (1981). Generalized descent for global optimization. J. Optim. Theory Appl..

[25] Sloane N.J.A., T.O.F. Inc. The On-Line Encyclopedia of Integer Sequences. http://oeis.org.

[26] Yang X.S. (2010). A new metaheuristic bat-inspired algorithm. Nature Inspired Cooperative Strategies for Optimization (NICSO 2010).

[27] Yang X.S., Slowik A. (2020). Bat algorithm. Swarm Intelligence Algorithms.

[28] Yang X.S., Deb S. Cuckoo search via Lévy flights. Proceedings of the 2009 World Congress on Nature & Biologically Inspired Computing (NaBIC).

[29] Yang X.S., Slowik A. (2020). Cuckoo Search Algorithm. Swarm Intelligence Algorithms: A Tutorial.

[30] Price K., Storn R.M., Lampinen J.A. (2006). Differential Evolution: A Practical Approach to Global Optimization.

[31] Yang X.S. (2009). Firefly Algorithms for Multimodal Optimization. Stochastic Algorithms: Foundations and Applications.

[32] Whitley D. (1994). A genetic algorithm tutorial. Stat. Comput..

[33] Mirjalili S., Mirjalili S.M., Lewis A. (2014). Grey Wolf Optimizer. Adv. Eng. Softw..

[34] Heidari A.A., Mirjalili S., Faris H., Aljarah I., Mafarja M., Chen H. (2019). Harris hawks optimization: Algorithm and applications. Future Gener. Comput. Syst..

[35] Rao R. (2016). Jaya: A simple and new optimization algorithm for solving constrained and unconstrained optimization problems. Int. J. Ind. Eng. Comput..

[36] Mirjalili S. (2015). Moth-flame optimization algorithm: A novel nature-inspired heuristic paradigm. Knowl.-Based Syst..

[37] Mirjalili S., Mirjalili S.M., Hatamlou A. (2015). Multi-Verse Optimizer: A nature-inspired algorithm for global optimization. Neural Comput. Appl..

[38] Kennedy J., Eberhart R. Particle swarm optimization. Proceedings of the ICNN’95—International Conference on Neural Networks.

[39] Mirjalili S. (2016). SCA: A sine cosine algorithm for solving optimization problems. Knowl.-Based Syst..

[40] Mirjalili S., Gandomi A.H., Mirjalili S.Z., Saremi S., Faris H., Mirjalili S.M. (2017). Salp Swarm Algorithm: A bio-inspired optimizer for engineering design problems. Adv. Eng. Softw..

[41] Mirjalili S., Lewis A. (2016). The Whale Optimization Algorithm. Adv. Eng. Softw..

[42] Faris H., Aljarah I., Mirjalili S., Castillo P.A., Guervós J.J.M. EvoloPy: An Open-Source Nature-Inspired Optimization Framework in Python. Proceedings of the 8th International Joint Conference on Computational Intelligence (IJCCI 2016).

[43] Ochoa G., Malan K.M., Blum C. (2021). Search trajectory networks: A tool for analysing and visualising the behaviour of metaheuristics. Appl. Soft Comput..

[44] Liang J.J., Qu B.Y., Suganthan P.N. (2013). Problem Definitions and Evaluation Criteria for the CEC 2014 Special Session and Competition on Single Objective Real-Parameter Numerical Optimization.

[45] Liang J., Qu B., Suganthan P., Chen Q. (2014). Problem Definitions and Evaluation Criteria for the CEC 2015 Competition on Learning-Based Real-Parameter Single Objective Optimization.

[46] Gao X., Alvo M., Chen J., Li G. (2008). Nonparametric multiple comparison procedures for unbalanced one-way factorial designs. J. Stat. Plan. Inference.

[47] Gao X., Alvo M. (2008). Nonparametric multiple comparison procedures for unbalanced two-way layouts. J. Stat. Plan. Inference.

[48] Massey F.J. (1951). The Kolmogorov-Smirnov Test for Goodness of Fit. J. Am. Stat. Assoc..

